# Machine learning-based derivation and validation of three immune phenotypes for risk stratification and prognosis in community-acquired pneumonia: a retrospective cohort study

**DOI:** 10.3389/fimmu.2024.1441838

**Published:** 2024-07-24

**Authors:** Qiangqiang Qin, Haiyang Yu, Jie Zhao, Xue Xu, Qingxuan Li, Wen Gu, Xuejun Guo

**Affiliations:** ^1^ Department of Respiratory Medicine, Xinhua Hospital, Shanghai Jiaotong University School of Medicine, Shanghai, China; ^2^ Department of Hematology, Xinhua Hospital, Shanghai Jiaotong University School of Medicine, Shanghai, China; ^3^ Department of Respiratory and Critical Care Medicine, The Second Hospital of Jilin University, Changchun, Jilin, China

**Keywords:** community-acquired pneumonia, immune phenotype, machine learning, unsupervised clustering, risk stratification

## Abstract

**Background:**

The clinical presentation of Community-acquired pneumonia (CAP) in hospitalized patients exhibits heterogeneity. Inflammation and immune responses play significant roles in CAP development. However, research on immunophenotypes in CAP patients is limited, with few machine learning (ML) models analyzing immune indicators.

**Methods:**

A retrospective cohort study was conducted at Xinhua Hospital, affiliated with Shanghai Jiaotong University. Patients meeting predefined criteria were included and unsupervised clustering was used to identify phenotypes. Patients with distinct phenotypes were also compared in different outcomes. By machine learning methods, we comprehensively assess the disease severity of CAP patients.

**Results:**

A total of 1156 CAP patients were included in this research. In the training cohort (n=809), we identified three immune phenotypes among patients: Phenotype A (42.0%), Phenotype B (40.2%), and Phenotype C (17.8%), with Phenotype C corresponding to more severe disease. Similar results can be observed in the validation cohort. The optimal prognostic model, SuperPC, achieved the highest average C-index of 0.859. For predicting CAP severity, the random forest model was highly accurate, with C-index of 0.998 and 0.794 in training and validation cohorts, respectively.

**Conclusion:**

CAP patients can be categorized into three distinct immune phenotypes, each with prognostic relevance. Machine learning exhibits potential in predicting mortality and disease severity in CAP patients by leveraging clinical immunological data. Further external validation studies are crucial to confirm applicability.

## Introduction

Community-acquired pneumonia (CAP) is an acute parenchymal lung infection caused by a variety of microorganisms outside the hospital. Despite advancements in rapid diagnostic testing, novel treatment options, and vaccine development, CAP continues to be one of the predominant causes of hospitalization, morbidity, and mortality globally (1). Severe community-acquired pneumonia (SCAP) is presently defined as the condition of patients requiring admission to the Intensive Care Unit (ICU) for mechanical ventilation (MV) or intensive respiratory or vasopressor support (IRVS) (2). Among 7,449 patients enrolled in the United States between 2014 and 2016, the 30-day mortality rate for SCAP was 6% (3). Consequently, the prompt identification and immediate management of SCAP are crucial for reducing its mortality rate. Presently, numerous methods are employed to evaluate the severity of CAP, primarily relying on established scores and guidelines. Nonetheless, these methods exhibit multiple limitations that impede their utility as clinical decision support tools (4–6).

In recent decades, machine learning (ML) algorithms have shown better performance in predicting various diseases or clinical conditions. Research has consistently illustrated the efficacy of ML in managing critically ill patients by predicting length of stay, risk of ICU readmission, and mortality rates. Recently, Jeon Et al. established that ML models significantly outperform traditional severity-of-illness scoring systems in predicting ICU mortality among patients with severe pneumonia (7). Xu et al. found that the ML model based on available clinical features is feasible and effective in predicting adverse outcomes such as mortality inCAP patients and ICU admission (8).

The clinical manifestations of CAP are highly variable. As a result, patients with CAP who are hospitalized present with a wide range of clinical symptoms, vital signs, and laboratory findings. Previously, Stefano Aliberti et al. divided patients into three different clinical phenotypes based on the presence or absence of acute respiratory failure and severe sepsis at admission, which showed significant differences in mortality (9). As infections advance, a range of resident and mobilized immune cells are activated to combat the invading pathogens. Research indicates that both the inflammatory response and immune regulation are pivotal in the pathogenesis of SCAP and acute respiratory distress syndrome (ARDS) (10). However, to date, limited studies have explored the immune phenotypes associated with CAP and their correlation with patient clinical outcomes. Therefore, we hypothesize that distinct clusters of characteristics present in CAP patients at admission may form identifiable subgroups or phenotypes, potentially signaling disparate prognoses for the illness and serving a vital function in the early detection of SCAP. This study sought to ascertain if immune phenotypes in patients with CAP can be identified using immunological data, to evaluate their correlation with prognosis, and to predict the likelihood of SCAP.

## Methods

### Study design

**Figure 1:**
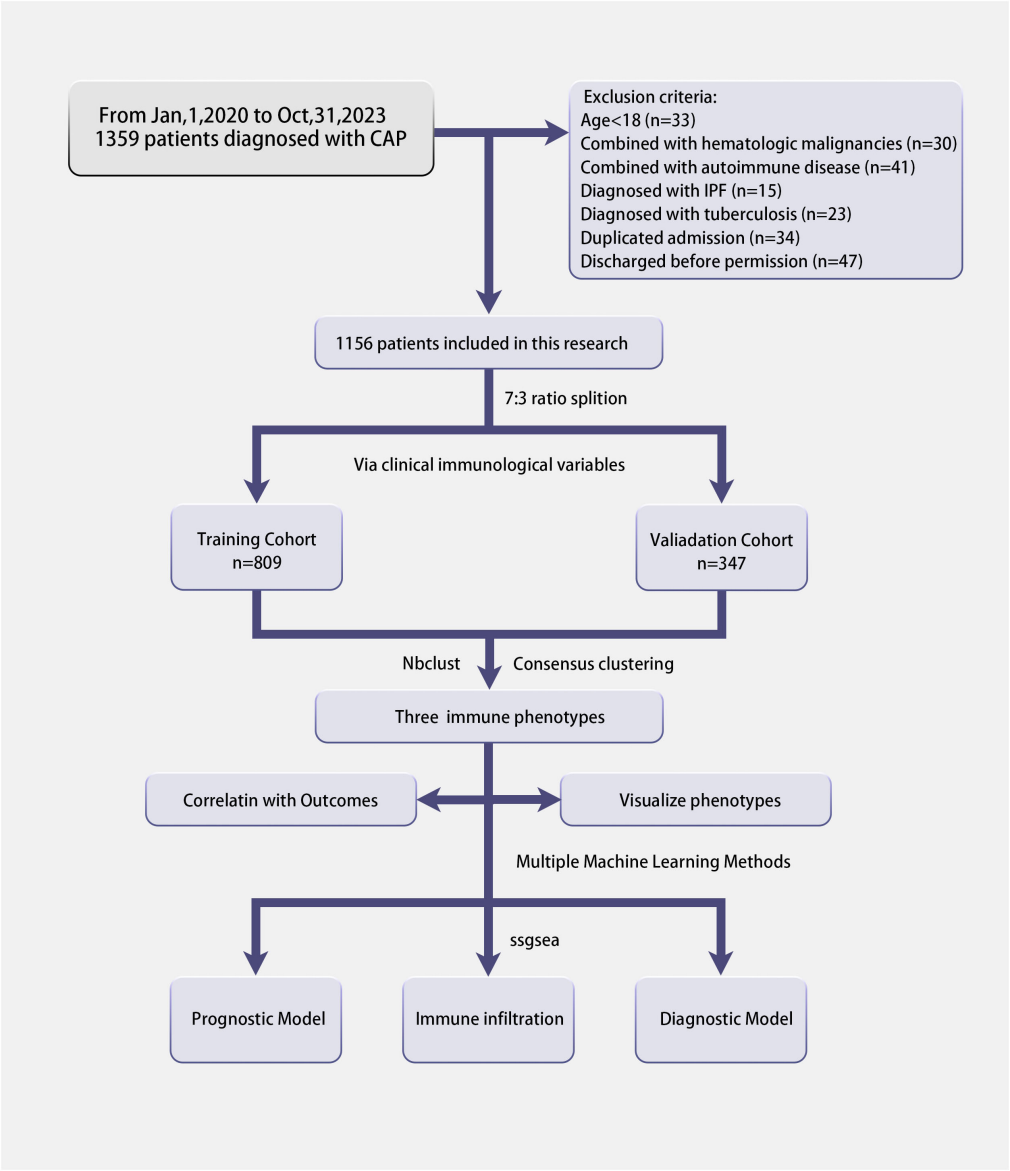
The flowchart of this research.

In this research, electronic health records of patients diagnosed with CAP admitted to the Respiratory and Critical Care department of Xin Hua Hospital Affiliated to Shanghai Jiao Tong University School of Medicine between January 1, 2020 and October 31, 2023 were retrospectively collected. All patients incorporated in this research were required to meet the diagnostic criteria of CAP and to have blood samples collected within the first 24 hours of admission. However, the study excluded patients who met any of the following exclusion criteria: (1) age under 18 years; (2) diagnosis of an autoimmune or hematologic malignancy; (3) a subsequent diagnosis of conditions such as pulmonary tuberculosis or idiopathic pulmonary fibrosis; and (4) those who declined further treatment or were transferred to another hospital. (see **Figure 1**, **Supplementary Figure S1** for details). Additionally, in this study, we exclusively consider data from the initial hospital admission for individuals who experienced multiple admissions (11). Vital signs(heart rate, systolic blood pressure and diastolic blood pressure, temperature, respiratory rate and mentation), demographic information(age, sex, height, weight), laboratory indicators(WBC, Neutrophil cell count, IgA, IgE, IgM, IL-6, IL-8, CD3, CD4, CD8, etc.) were collected within 24h after admission, and other variables (days from symptom onset, chief complaint, length of stay, application of assisted ventilation, and clinical outcome) were also extracted after patients discharged. Upon applying our predefined inclusion and exclusion criteria, we successfully enrolled a cohort of 1,165 eligible patients for this study.

### Candidate variables for clustering and phenotyping

Patients were randomly allocated to the training and validation cohorts in a ratio of 7:3 to ensure reproducibility and consistency in the modeling outcomes (12). And a meticulously curated dataset comprising various laboratory indicators has been compiled for this analysis. When addressing missing values, we employed two distinct strategies. For variables with less than 20% missing data, imputation was performed using the Multivariate Imputation by Chained Equations (MICE) package (13). Conversely, variables with 20% or more missing data were excluded entirely to preserve the integrity and robustness of the study (14). This methodological approach refined our dataset to encompass 79 laboratory indicators. Moreover, given the study’s emphasis on exploring the immune phenotype of community-acquired pneumonia, we cumulatively identified 31 immunological laboratory variables as the focal point for clustering analysis. To identify commonalities among different patients based on laboratory examinations, we applied the unsupervised ‘consensus clustering’ algorithm within the training cohort to ascertain the optimal number of clusters. Subsequently, we corroborated the findings in both the validation cohort and the meta-cohort. Additionally, to verify the integrity of the clustering process, our dataset underwent analysis using the NBclust (15)clustering algorithm. Additionally, we employed an alluvial plot to visualize the discrepancies between conventional grading systems and our machine learning approach. Lastly, we presented chord diagrams to illustrate the associations between laboratory indicators and clinical immune phenotypes.

### Definitions and clinical outcomes

CAP patients were classified into three distinct immune phenotypes (Phenotype A, Phenotype B, and Phenotype C). Utilizing prior knowledge and clinical expertise, indicators such as the necessity for mechanical ventilation, admission to the intensive care unit, or mortality due to the disease were employed as surrogate markers of SCAP in this investigation. The primary outcome was the in-hospital mortality rate. Secondary outcomes included the 28-day mortality rate, the likelihood of progressing to severe pneumonia, length of stay, days of ventilation, days in the ICU, ICU-free days, and the probability of respiratory support. Upon stratification of pneumonia patients into three immune phenotypes, we assessed differences in all-cause in-hospital mortality rates across the meta, training, and validation cohorts. Simultaneously, we examined the 28-day mortality rate and the risk of severe disease progression within these cohorts. This approach facilitated a detailed evaluation of mortality outcomes associated with different immune phenotypes in community-acquired pneumonia.

### Integrated machine learning based model construction and evaluation

To assess the predictive performance of machine learning (ML) methods based on Zaoqu liu’s framework (16), nine algorithms were employed for patient prognosis prediction in both the training and validation cohorts. The model with the highest average concordance index (C-index) across these cohorts will be considered the most effective. Additionally, this study expanded its methodology to include thirteen ML algorithms for forecasting the risk of severity in CAP patients, specifically: Lasso, Ridge, Elastic Net (Enet), Stepwise GLM (Stepglm), Support Vector Machine (SVM), Gradient Boosting Machine (GBM), Linear Discriminant Analysis (LDA), Partial Least Squares and Logistic Regression Model (plsRglm), Random Forest, and Naive Bayes (17–19). Consistently, the model that exhibits superior performance across both cohorts will be identified as the optimal model. To guarantee the robustness and reliability of our models, we meticulously selected only those comprising more than five variables. This strategy enabled clinicians to concentrate on the most informative and stable combinations of predictors. Moreover, we incorporated the publicly accessible dataset GSE188309 (20), which concentrates on community-acquired pneumonia, into our analysis to ascertain potential immune infiltration from a transcriptomic perspective.

### Association between clinical immune phenotype and traditional scoring system

Generally, CURB-65 and PSI scoring systems were frequently utilized in clinical settings to evaluate the severity of pneumonia patients with CAP. However, recent studies have highlighted significant limitations within these systems (21, 22). In response, we developed a model utilizing machine learning techniques and evaluated its predictive accuracy using the Receiver Operating Characteristic (ROC) curve, in comparison to traditional scoring systems.

### Statistical analysis

In this study, the Shapiro-Wilk test was utilized to assess the normality of continuous variables prior to the formal analysis. For comparisons, the Mann–Whitney U-test was applied to non-normally distributed continuous variables, while the Student’s t-test was used for those with a normal distribution, as appropriate. For categorical variables, the Kruskal-Wallis test or the Chi-square test was employed where relevant. Continuous variables were presented as mean ± SD for normally distributed data and as median (interquartile range, IQR) for non-normally distributed data. Categorical variables were reported as frequencies and percentages. All data generation, processing, statistical analyses, and plotting were performed using R software version 4.2.0. A p-value of less than 0.05 was considered statistically significant, although this was not explicitly mentioned in the report.

### IRB statement

Approval was obtained from the Institutional Review Board (IRB) of Xinhua Hospital, Shanghai Jiao Tong University School of Medicine, Shanghai, China, and a waiver of consent was granted because the study used electronic medical record data and blood test results from normal clinical visits (Approval Number: XHEC-C-2024-026-1; Approval Date: 2024-03-19; Study Title: Clinical Study Based on Comprehensive Multi-omics Analysis of Peripheral Blood for Community Acquired Pneumonia). All procedures were followed in accordance with the IRBs standards on human experimentation and the Helsinki Declaration of 1975.

## Results

### Baseline characteristics of CAP patients

**Figure 2:**
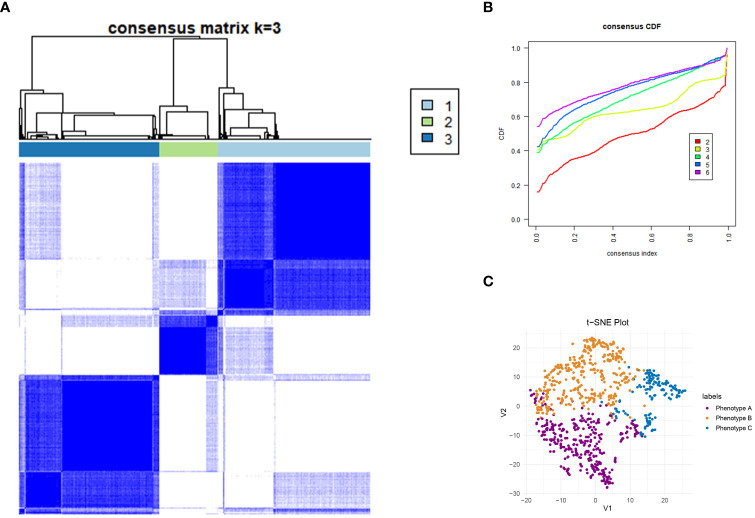
Consensus Clustering and visualization. **(A)** Identification of three immune phenotypes of community acquired pneumonia (CAP) patients by consensus clustering. **(B)** Cumulative distribution function (CDF) curve illustrated consensus distribution for each phenotype. **(C)** T-distributed stochastic neighbor embedding (t-SNE) method successfully divided CAP patients into three distinct immune phenotypes. The purple dot represent patients belong to phenotype A. Patients with phenotype B are represented by a yellow dot, and those with phenotype C by a blue dot. CAP, community acquired pneumonia; t-SNE, T-distributed stochastic neighbor embedding; CDF, Cumulative distribution function.

**Figure 3:**
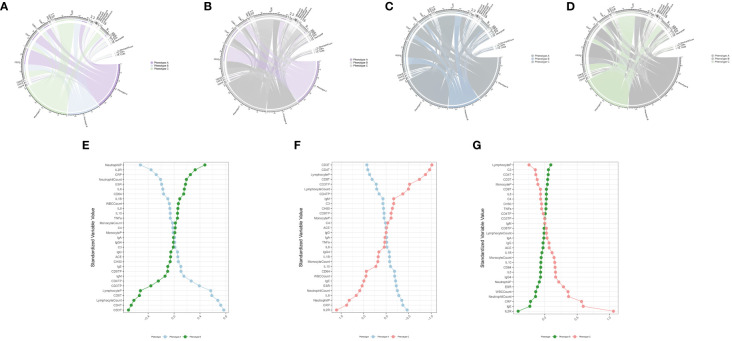
Association and variation between clinical immunological indicators and three phenotypes. Chord diagram **(A–D)** of the association between clinical immunological variables and each phenotype in training cohort. Different phenotypes were shown in different colors: phenotype A is purple, phenotype B is blue, and phenotype C is green. Rank plot **(E–G)** of variable mean among various phenotypes in training cohort. Variables were normalized by mean and standard error.

This study reviewed the records of 12,000 individuals discharged from the Respiratory Department of Xinhua Hospital between January 1, 2020, and October 31, 2023. Of these, 1,379 were diagnosed with CAP. Following the application of exclusion criteria, 223 patients were omitted from the study. Consequently, a cohort of 1,156 CAP patients was established for inclusion in the research. Patients were allocated into two groups: a training cohort consisting of 809 patients and a validation cohort of 347 patients, using a randomization ratio of 7:3. **Table 1** presents the baseline characteristics of the combined meta-cohort, along with those of the training and validation cohorts separately. Among the participants, 8 required treatment with an invasive ventilator, 96 received non-invasive ventilation, and 46 underwent therapy with high-flow nasal cannula. In this study, 53 patients succumbed to their conditions, while 239 required admission to the intensive care unit. The median hospital stay for the meta cohort was 9 days, with an interquartile range (IQR) of 7–14 days. The three most frequent symptoms among the patients were cough (76%, n=880), fever (54%, n=626), and sputum production (52%, n=600). Consistent with prior research (23, 24), the most prevalent comorbidities included hypertension (40%, n=467) and type 2 diabetes mellitus (22%, n=252). To determine the latent immune phenotypes of CAP, the “Consensus Cluster Plus” package was utilized for consensus clustering across the training, validation, and meta cohorts. This analysis identified three distinct immune phenotypes within each cohort, as demonstrated in **Figures 2A, B**, **Supplementary Figures S3A, B**, and **S4A, B**, indicating that a tripartite classification was most suitable for the data. The “nbclust” package (15, 25) was routinely utilized for unsupervised clustering to ascertain the optimal number of clusters. In agreement with the majority rule, three distinct clusters were deemed to be ideal for all cohorts, as depicted in the **Supplementary Materials** (**Supplementary Figures S2A**, **S3C**, and **S4C**). Grounded on these findings and informed by prior knowledge, a tripartite classification of phenotypes was determined to be most appropriate. The results of clustering were visualized by t-distributed stochastic neighbor embedding (t-SNE), Principal Component Analysis (PCA) and Uniform Manifold Approximation and Projection (UMAP) plot, as shown in **Figure 2C**, **Supplementary Figures S2B, C**, **S3D–F**, and **S4D–F**. The baseline characteristics of each cohort are presented in **Table 2** and **Supplementary Tables S1**, **S2**. The three distinct immune phenotypes varied in size, ranging from 16.8% to 43.4% of the cohorts, and demonstrated differences in clinical presentations and patterns of organ dysfunction. These variances are detailed in **Supplementary Tables S1**, **S2**, and **Figures 3A–D**, **Supplementary Figures S7A–D**, and **S8A–D**. Within the training cohort, patients with CAP were grouped into three phenotypes based on distinct laboratory features. Phenotype A patients exhibited elevated levels of prealbumin. In contrast, phenotype B was characterized by reduced IgG4, triglycerides, and uric acid levels. Patients classified as phenotype C tended to be older males with a higher likelihood of being admitted to the ICU. Variations in laboratory indicators were apparent among the three immune phenotypes. According to the standardized mean difference between phenotypes (**Figures 3E–G**), phenotype A patients showed fewer laboratory abnormalities and less evidence of organ dysfunction. Conversely, phenotype B patients had indicators suggestive of renal dysfunction, while those in phenotype C were more likely to display increased inflammatory markers (such as IL-2R, IL-8, IL-6), alongside reduced immunologic parameters of inflammation (e.g., CD3, CD4, CD8), lower albumin levels, and elevated body temperatures. Patients classified as Phenotype B tend to demonstrate moderate abnormalities in their laboratory tests. Relative to Phenotype A, those with Phenotype B show elevated levels of neutrophils, C-reactive protein (CRP), and erythrocyte sedimentation rate (ESR). In addition, there is a concurrent suppression of lymphocytes and their subsets. The levels of IgG4 do not vary significantly between these phenotypes. Patients identified as Phenotype C also manifest a similar pattern, with increased inflammatory markers (IL-2R, neutrophil count, and ESR) and decreased immunological indicators (CD3+CD4+CD8+ lymphocytes); however, IgG, IgA, and TNF-α levels remain statistically unchanged. When drawing comparisons between Phenotypes B and C, a rise in IL-2R, CRP, ESR, and IgE is noted, along with a reduction in lymphocyte-related indicators. These differential markers underscore their importance in phenotype classification and, indirectly, the robustness of this classification scheme. Further corroboration of these findings is evident in the validation and meta cohorts, as depicted in **Supplementary Figures S7E–G** and **S8E–G**.

**Table 1:** Characteristics of the study population and outcomes of community acquired pneumonia patients.

Variables	Total (n = 1156)	test (n = 347)	train (n = 809)	P Value
Demographics				
Sex, n (%)				0.715
Female	492 (43)	151 (44)	341 (42)	
Male	664 (57)	196 (56)	468 (58)	
Age (years), median (IQR)	65 (54, 73)	66 (58, 73)	65 (53, 73)	0.534
Height (cm), median (IQR)	166 (160, 172)	167 (160, 173)	166 (160, 172)	0.783
Weight (kg), median (IQR)	64 (56, 73)	64.75 (57, 73.08)	64 (55, 73)	0.356
Mentation, n (%)				0.721
1	1141 (99)	343 (99)	798 (99)	
2	4 (0)	1 (0)	3 (0)	
3	5 (0)	2 (1)	3 (0)	
4	2 (0)	0 (0)	2 (0)	
5	1 (0)	0 (0)	1 (0)	
6	0 (0)	0 (0)	0 (0)	
7	2 (0)	0 (0)	2 (0)	
8	1 (0)	1 (0)	0 (0)	
Vital signs				
Fever peak, median (IQR)	38.1 (37.3, 39)	38 (37.3, 39)	38.1 (37.3, 39)	0.661
Temperature admission, median (IQR)	37 (36.6, 37.7)	37 (36.7, 37.7)	37 (36.6, 37.7)	0.568
Tmax during the course, median (IQR)	37.4 (37.1, 38)	37.3 (37.05, 38)	37.4 (37.1, 38)	0.405
Heart rate, median (IQR)	92 (83, 101)	90 (82, 101)	92 (83, 101)	0.202
Respiration Rate, median (IQR)	20 (18, 20)	20 (19, 20)	20 (18, 20)	0.204
Systolic blood pressure, median (IQR)	132 (120, 149)	131 (120, 148.75)	133 (120, 149)	0.684
Diastolic blood pressure, median (IQR)	79 (71, 87)	79 (72, 86)	78 (70, 88)	0.762
CURB-65 score, n (%)				0.883
0	452 (39)	130 (37)	322 (40)	
1	444 (38)	137 (39)	307 (38)	
2	222 (19)	70 (20)	152 (19)	
3	37 (3)	10 (3)	27 (3)	
4	1 (0)	0 (0)	1 (0)	
PSI score, median (IQR)	70 (53, 89)	71 (54, 88)	69 (52, 90)	0.274
Chief Complaint				
Cough, n (%)				0.339
No	276 (24)	76 (22)	200 (25)	
Yes	880 (76)	271 (78)	609 (75)	
Sputum, n (%)				0.281
No	556 (48)	158 (46)	398 (49)	
Yes	600 (52)	189 (54)	411 (51)	
Chest Pain, n (%)				0.792
No	1081 (94)	326 (94)	755 (93)	
Yes	75 (6)	21 (6)	54 (7)	
Dyspnea, n (%)				0.469
No	874 (76)	257 (74)	617 (76)	
Yes	282 (24)	90 (26)	192 (24)	
Chest Tightness, n (%)				0.329
No	1025 (89)	313 (90)	712 (88)	
Yes	131 (11)	34 (10)	97 (12)	
Fever, n (%)				0.661
No	530 (46)	163 (47)	367 (45)	
Yes	626 (54)	184 (53)	442 (55)	
Fatigue, n (%)				0.364
No	1112 (96)	337 (97)	775 (96)	
Yes	44 (4)	10 (3)	34 (4)	
Consciousness Disorder, n (%)				0.207
No	1149 (99)	343 (99)	806 (100)	
Yes	7 (1)	4 (1)	3 (0)	
Difficulty Breathing, n (%)				0.706
No	1128 (98)	340 (98)	788 (97)	
Yes	28 (2)	7 (2)	21 (3)	
Hemoptysis, n (%)				0.364
No	1112 (96)	337 (97)	775 (96)	
Yes	44 (4)	10 (3)	34 (4)	
Comorbidity				
Respiratory system				
COPD, n (%)				0.537
No	1007 (87)	306 (88)	701 (87)	
Yes	149 (13)	41 (12)	108 (13)	
Bronchiectasis, n (%)				0.602
No	1088 (94)	329 (95)	759 (94)	
Yes	68 (6)	18 (5)	50 (6)	
Emphysema bullae, n (%)				1
No	1105 (96)	332 (96)	773 (96)	
Yes	51 (4)	15 (4)	36 (4)	
Pulmonary hypertension, n (%)				0.842
No	1126 (97)	337 (97)	789 (98)	
Yes	30 (3)	10 (3)	20 (2)	
Lung cancer, n (%)				0.602
No	1097 (95)	327 (94)	770 (95)	
Yes	59 (5)	20 (6)	39 (5)	
PE, n (%)				0.379
No	1116 (97)	338 (97)	778 (96)	
Yes	40 (3)	9 (3)	31 (4)	
Asthma, n (%)				0.188
No	1108 (96)	328 (95)	780 (96)	
Yes	48 (4)	19 (5)	29 (4)	
OSAHS, n (%)				0.225
No	1110 (96)	329 (95)	781 (97)	
Yes	46 (4)	18 (5)	28 (3)	
Bronchitis, n (%)				1
No	1136 (98)	341 (98)	795 (98)	
Yes	20 (2)	6 (2)	14 (2)	
Cardiovascular system				
Hypertension, n (%)				0.107
No	689 (60)	194 (56)	495 (61)	
Yes	467 (40)	153 (44)	314 (39)	
Atrial fibrillation, n (%)				0.08
No	1086 (94)	333 (96)	753 (93)	
Yes	70 (6)	14 (4)	56 (7)	
Coronary heart disease, n (%)				1
No	1026 (89)	308 (89)	718 (89)	
Yes	130 (11)	39 (11)	91 (11)	
Arrhythmia, n (%)				0.015
No	1005 (87)	315 (91)	690 (85)	
Yes	151 (13)	32 (9)	119 (15)	
Heart failure, n (%)				0.694
No	994 (86)	301 (87)	693 (86)	
Yes	162 (14)	46 (13)	116 (14)	
Digestive system				
Liver dysfunction, n (%)				1
No	992 (86)	298 (86)	694 (86)	
Yes	164 (14)	49 (14)	115 (14)	
Gastric cancer, n (%)				1
No	1146 (99)	344 (99)	802 (99)	
Yes	10 (1)	3 (1)	7 (1)	
Colorectal cancer, n (%)				0.132
No	1138 (98)	345 (99)	793 (98)	
Yes	18 (2)	2 (1)	16 (2)	
Esophageal cancer, n (%)				0.33
No	1151 (100)	347 (100)	804 (99)	
Yes	5 (0)	0 (0)	5 (1)	
Hepatitis, n (%)				0.163
No	1151 (100)	344 (99)	807 (100)	
Yes	5 (0)	3 (1)	2 (0)	
Urinary system				
Renal Insufficiency, n (%)				0.163
No	1076 (93)	329 (95)	747 (92)	
Yes	80 (7)	18 (5)	62 (8)	
Urological tumors, n (%)				0.573
No	1141 (99)	344 (99)	797 (99)	
Yes	15 (1)	3 (1)	12 (1)	
Kidney Stones, n (%)				0.473
No	1125 (97)	340 (98)	785 (97)	
Yes	31 (3)	7 (2)	24 (3)	
Endocrine system				
Diabetes, n (%)				0.065
No	904 (78)	259 (75)	645 (80)	
Yes	252 (22)	88 (25)	164 (20)	
Nervous system				
Senile dementia, n (%)				0.465
No	1147 (99)	343 (99)	804 (99)	
Yes	9 (1)	4 (1)	5 (1)	
Cerebral infarction, n (%)				1
No	1056 (91)	317 (91)	739 (91)	
Yes	100 (9)	30 (9)	70 (9)	
PD, n (%)				1
No	1144 (99)	344 (99)	800 (99)	
Yes	12 (1)	3 (1)	9 (1)	
History of mlignancy, n (%)				0.572
No	1042 (90)	309 (89)	733 (91)	
Yes	114 (10)	38 (11)	76 (9)	
Full Blood Count				
Hemoglobin (g/L), median (IQR)	125 (113, 135)	125 (116, 135.5)	125 (112, 135)	0.284
Neutrophil percent (%), median (IQR)	69 (58.9, 80.6)	69.8 (58.4, 80.3)	68.6 (59.3, 80.8)	0.789
Neutrophil count (*10^9/L), median (IQR)	4.43 (3.18, 6.75)	4.32 (3.1, 6.78)	4.46 (3.23, 6.72)	0.683
MCV (fl), median (IQR)	90.7 (88.3, 94)	90.8 (88.5, 93.95)	90.7 (88.1, 94)	0.435
MCH (pg), median (IQR)	30.3 (29.1, 31.4)	30.4 (29.3, 31.45)	30.2 (29.1, 31.4)	0.225
MCHC (g/L), median (IQR)	333 (325, 340)	334 (325, 341)	332 (325, 340)	0.295
MPV (fl), median (IQR)	9.4 (8.6, 10.2)	9.5 (8.7, 10.4)	9.3 (8.6, 10.2)	0.278
WBC Count (*10^9/L), median (IQR)	6.66 (5.18, 8.77)	6.61 (5.18, 8.62)	6.7 (5.18, 8.81)	0.925
Lymphocyte percent (%), median (IQR)	20.4 (11.6, 30)	20.1 (11.9, 29.65)	20.5 (11.2, 30)	0.86
Lymphocyte count (*10^9/L), median (IQR)	1.3 (0.86, 1.78)	1.34 (0.86, 1.78)	1.29 (0.87, 1.78)	0.481
Eosinophil percent (%), median (IQR)	1.5 (0.5, 2.9)	1.5 (0.5, 2.9)	1.5 (0.5, 2.8)	0.75
Monocyte percent (%), median (IQR)	6.7 (5.2, 8.53)	6.7 (5.2, 8.55)	6.8 (5.2, 8.5)	0.942
Monocyte count (*10^9/L), median (IQR)	0.44 (0.33, 0.61)	0.44 (0.32, 0.62)	0.44 (0.33, 0.61)	0.885
RDW (%), median (IQR)	13 (12.5, 13.6)	12.9 (12.4, 13.5)	13 (12.5, 13.6)	0.148
Arterial Blood Gas				
HCO3 (mmol/L), median (IQR)	25.4 (23.3, 27.4)	25.5 (23.35, 27.4)	25.3 (23.3, 27.3)	0.513
HCO3std (mmol/L), median (IQR)	25.3 (23.7, 26.9)	25.4 (23.7, 26.85)	25.2 (23.7, 26.9)	0.72
pCO2 (kPa), median (IQR)	5.21 (4.72, 5.66)	5.23 (4.75, 5.66)	5.21 (4.7, 5.65)	0.352
pH, median (IQR)	7.42 (7.4, 7.45)	7.42 (7.4, 7.45)	7.42 (7.4, 7.45)	0.996
pO2 (kPa), median (IQR)	11.95 (10.3, 14.6)	11.7 (10.1, 14.15)	12.1 (10.5, 14.7)	0.061
TCO2 (mmol/L), median (IQR)	49.9 (44.98, 54.4)	50.1 (45.5, 54.45)	49.8 (44.7, 54.4)	0.556
Glu (mmol/L), median (IQR)	5.28 (4.66, 6.66)	5.3 (4.69, 6.76)	5.26 (4.64, 6.64)	0.264
Renal Function				
Cr (umol/L), median (IQR)	60 (51, 73)	58.8 (50, 72)	60 (51, 74)	0.141
BUN (mmol/L), median (IQR)	5.2 (4.02, 7)	5.2 (4.02, 7)	5.2 (4.02, 6.99)	0.963
GFR (mL/min per1.75m^2), median (IQR)	104.72 (84.94, 125.35)	106.05 (87.31, 125.96)	104.37 (84.2, 124.44)	0.522
UA (umol/L), median (IQR)	269 (213, 338)	264 (217, 313.5)	269 (210, 345)	0.354
ACE (U/L), median (IQR)	23.5 (17.28, 30.2)	23.4 (17, 29.9)	23.6 (17.5, 30.5)	0.97
Blood lipids				
LDL-C (mmol/L), median (IQR)	2.41 (1.89, 2.97)	2.53 (1.94, 3.08)	2.38 (1.87, 2.95)	0.05
TG (mmol/L), median (IQR)	1.04 (0.77, 1.41)	1.08 (0.79, 1.43)	1.02 (0.76, 1.4)	0.303
ApoE (mg/dL), median (IQR)	3.6 (2.9, 4.6)	3.7 (2.9, 4.5)	3.6 (2.9, 4.7)	0.846
Coagulation				
D-Dimer (mg/L), median (IQR)	0.61 (0.34, 1.16)	0.61 (0.32, 1.14)	0.61 (0.34, 1.17)	0.513
TT (s), median (IQR)	13.7 (12.8, 14.7)	13.8 (12.8, 14.65)	13.7 (12.8, 14.8)	0.773
APTT (s), median (IQR)	11.9 (11, 12.9)	11.8 (11, 12.9)	11.9 (11.1, 13)	0.31
INR, median (IQR)	1.05 (0.97, 1.14)	1.04 (0.97, 1.14)	1.05 (0.97, 1.15)	0.346
PTT (s), median (IQR)	30.9 (28.5, 33.4)	30.5 (28.05, 32.95)	31.1 (28.7, 33.6)	0.024
ATA (%), median (IQR)	83 (72, 93)	84 (71.5, 95)	83 (72, 93)	0.258
Inflammatory Cytokine				
IL-10 (pg/mL), median (IQR)	5 (5, 5)	5 (5, 5)	5 (5, 5)	0.085
IL-1B (pg/mL), median (IQR)	5 (5, 9.76)	5 (5, 9.93)	5 (5, 9.72)	0.874
IL-2R (U/mL), median (IQR)	573.5 (409, 880.5)	569 (397, 851)	582 (412, 899)	0.218
IL-6 (pg/mL), median (IQR)	6.97 (3.09, 19.2)	6.83 (2.91, 18.4)	7.18 (3.18, 19.4)	0.553
IL-8 (pg/mL), median (IQR)	26.3 (14.67, 62.12)	28.2 (14.8, 66.35)	25.6 (14.4, 61)	0.628
TNF-α (pg/mL), median (IQR)	14.65 (8.97, 28.52)	14.9 (9.14, 29.6)	14.6 (8.91, 28.4)	0.441
IgG4 (g/L), median (IQR)	0.5 (0.26, 0.9)	0.48 (0.25, 0.86)	0.5 (0.27, 0.93)	0.541
Electrolytes				
P (mmol/L), median (IQR)	1.08 (0.93, 1.22)	1.08 (0.95, 1.21)	1.07 (0.92, 1.22)	0.394
Cl (mmol/L), median (IQR)	105 (102, 107)	105 (102, 107)	105 (102, 107)	0.554
Mg (mmol/L), median (IQR)	0.92 (0.85, 0.97)	0.92 (0.85, 0.97)	0.92 (0.85, 0.98)	0.356
Potassium (mmol/L), median (IQR)	3.96 (3.69, 4.21)	3.95 (3.69, 4.19)	3.96 (3.68, 4.22)	0.748
Ca (mmol/L), median (IQR)	2.08 (1.99, 2.16)	2.09 (1.99, 2.15)	2.08 (1.99, 2.16)	0.969
Sodium (mmol/L), median (IQR)	139 (136, 141)	139 (136, 141)	139 (136, 141)	0.277
Inflammation Measurements				
CRP (mg/L), median (IQR)	24 (4, 76)	21 (3, 68.5)	25 (4, 78)	0.261
PCT(ng/mL), median (IQR)	0.05 (0.04, 0.15)	0.05 (0.04, 0.14)	0.05 (0.04, 0.15)	0.149
ESR (mm/h), median (IQR)	41.5 (21, 70)	42 (21, 70)	41 (21, 69)	0.875
Myocardial Enzyme				
CK-MB (U/L), median (IQR)	5 (3, 7)	5 (3.1, 8)	5 (3, 7)	0.983
cTnI (ng/ml), median (IQR)	0.01 (0, 0.01)	0.01 (0, 0.01)	0.01 (0, 0.01)	0.291
CK (U/L), median (IQR)	63 (41, 99.25)	58 (41, 88)	65 (41, 103)	0.123
α-HBDH (U/L), median (IQR)	142 (113.75, 173)	141 (114.5, 173)	142 (113, 173)	0.711
LDH (U/L), median (IQR)	212 (176, 264)	214 (176, 262)	211 (176, 264)	0.702
Cell immunity				
CD3T (%), median (IQR)	70.5 (62.97, 77.29)	69.95 (61.91, 77.15)	70.67 (63.58, 77.37)	0.282
CD4T (%), median (IQR)	41.8 (34.47, 48.45)	41.01 (33.45, 48.59)	41.99 (35.02, 48.31)	0.431
CD8T (%), median (IQR)	24.13 (18.48, 30.66)	24.07 (18.59, 30.9)	24.22 (18.43, 30.56)	0.915
CD3T (Cells/uL), median (IQR)	932.35 (567.65, 1285.11)	932.52 (557.31, 1291.49)	932.18 (578.03, 1284.19)	0.827
CD4T (Cells/uL), median (IQR)	532.42 (320.8, 785.7)	515.96 (305.94, 800.39)	537.39 (332.43, 776.03)	0.742
CD64 infection index	0.84 (0.43, 2.06)	0.89 (0.43, 2.06)	0.84 (0.42, 2.06)	0.892
CD8T (Cells/uL), median (IQR)	314.12 (185.76, 461.07)	316.69 (185.76, 461.9)	313.71 (185.79, 459.57)	0.805
Humoral Immunity				
Ig A (g/L), median (IQR)	2.5 (1.86, 3.31)	2.49 (1.85, 3.29)	2.5 (1.86, 3.33)	0.792
Ig E (IU/mL), median (IQR)	59.45 (21.7, 197.25)	62.3 (22.15, 184.5)	57.9 (21.2, 211)	0.921
Ig G (g/L), median (IQR)	12.3 (10.5, 14.5)	12 (10.4, 14.2)	12.4 (10.6, 14.6)	0.122
Ig M (g/L), median (IQR)	0.86 (0.62, 1.21)	0.89 (0.58, 1.22)	0.86 (0.62, 1.2)	0.949
Liver Function				
GGT (U/L), median (IQR)	29 (18, 53)	28 (19, 49)	29 (18, 55)	0.583
Alb (g/L), median (IQR)	35.8 (32.1, 39)	35.9 (32.3, 38.8)	35.8 (32, 39.1)	0.977
AST (U/L), median (IQR)	20 (13, 34)	22 (13, 34)	20 (12, 34)	0.356
ALT (U/L), median (IQR)	21 (16, 31)	20 (16, 29.5)	21 (16, 31)	0.147
ALP (U/L), median (IQR)	75 (61, 95)	76 (60.5, 95)	75 (61, 94)	0.79
PA (mg/L), median (IQR)	158 (111, 206)	156 (116, 206)	159 (109, 207)	0.597
TB (umol/L), median (IQR)	8.7 (6.4, 11.4)	8.6 (6.2, 10.9)	8.7 (6.5, 11.5)	0.311
TP (g/L), median (IQR)	63.9 (59.5, 67.8)	63.5 (59.2, 67.8)	64 (59.8, 67.8)	0.471
FIB (g/L), median (IQR)	4.05 (3.24, 4.94)	3.9 (3.24, 4.95)	4.06 (3.24, 4.92)	0.903
Complement system				
C3 (g/L), median (IQR)	1.17 (1, 1.35)	1.17 (1.01, 1.34)	1.17 (1, 1.36)	0.976
C4 (g/L), median (IQR)	0.29 (0.23, 0.37)	0.29 (0.23, 0.37)	0.29 (0.23, 0.37)	0.898
CH50 (U/mL), Mean ± SD	49.59 _ 15.19	49.31 _ 15.27	49.71 _ 15.16	0.685
Respiratory support				
HFNC, n (%)				0.668
No	1110 (96)	335 (97)	775 (96)	
Yes	46 (4)	12 (3)	34 (4)	
NIMV, n (%)				0.941
No	1060 (92)	319 (92)	741 (92)	
Yes	96 (8)	28 (8)	68 (8)	
IMV, n (%)				1
No	1148 (99)	345 (99)	803 (99)	
Yes	8 (1)	2 (1)	6 (1)	
Clinical Outcomes				
ICU duration (days), median (IQR)	0 (0, 0)	0 (0, 0)	0 (0, 0)	0.444
Ventilation duration (days), median (IQR)	0 (0, 0)	0 (0, 0)	0 (0, 0)	0.44
Length of stay, median (IQR)	9 (7, 14)	10 (7, 14)	9 (7, 14)	0.372
Inpatient Outcome, n (%)				0.46
Alive	1103 (95)	334 (96)	769 (95)	
Dead	53 (5)	13 (4)	40 (5)	
ICU free days (days), median (IQR)	8 (4, 11)	8 (5, 12)	7 (4, 10)	0.12
Outcome at 28 days, n (%)				0.433
Alive	1117 (97)	338 (97)	779 (96)	
Dead	39 (3)	9 (3)	30 (4)	
Days from symptom onset (days), median (IQR)	10 (6, 14)	10 (5, 14)	10 (6, 14)	0.811
ICU admission, n (%)				0.502
No	917 (79)	280 (81)	637 (79)	
Yes	239 (21)	67 (19)	172 (21)	

COPD, Chronic obstructive pulmonary disease; OSAHS, Obstructive sleep apnea hypopnea syndrome; MCV, Mean corpuscular volume; MCH, Mean corpuscular hemoglobin; MCHC, Mean corpuscular hemoglobin concentration; WBC, White bold cell; MPV, Mean platelet volume; RDW, Red blood cell distribution width; HCO3, Carbonic acid hydrogen radical; HCO3std, Standard bicarbonate; pCO2, Partial pressure of carbon dioxide; Ph, Potential of hydrogen; pO2, Partial pressure of oxygen; TCO2, Total carbon dioxide; Glu, Glucose; Cr,Creatinine; BUN, Blood urea nitrogen; GFR, Glomerular Filtration Rate; UA, Urine Acid; ACE, Angiotensin-Converting Enzyme; LDL-C, Low-Density Lipoprotein Cholesterol; TG, Triglyceride; ApoE, Apolipoprotein E; TT, Thrombin time; APTT, Activated partial thromboplastin time; INR, International normalized ratio; PTT, Partial thromboplastin time; ATA, Antithrombin Activity; IL-10, Lnterleukin-10; IL-1B, Lnterleukin-1B; IL-2R, Lnterleukin-2 Receptor; IL-6, Lnterleukin-6; IL-8, Lnterleukin-8; TNF-α, Tumor necrosis factor-alpha; IgG4, Immunoglobulin G4; CK-MB, Creatine kinase MB; cTnI, Cardiac troponin I; CK, Creatine kinase; α-HBDH, Alpha-hydroxybutyric dehydroge; LDH, Lactate dehydrogenase; Ig A, Immunoglobulin A; Ig E, Immunoglobulin E; Ig G, Immunoglobulin G; Ig M, Immunoglobulin M; GGT, γ-Glutamyl transferase GGT; Alb, Albumin; AST, Aspartate aminotransferase; ALT, Alanine aminotransferase; ALP, Alkaline phosphatase; PA, Prealbumin; TB, Total bilirubin; TP, Total Protein; FIB, Fibrinogen; C3, Complement C3; C4, Complement C4; CH50, 50% Hemolytic unit of Complement; HFNC, High Flow Nasal Cannula; NIMV, Noninvasive Mechanical Ventilation; IMV, Invasive Mechanical Ventilation.

**Table 2:** Characteristics and outcomes of community acquired pneumonia patients divided by immune phe Notypes in training cohort.

Phenotype	Phenotype A(n=322)	Phenotype B(n=351)	Phenotype C(n=136)	Total(n=809)	P value
Demographics					
Sex, n (%)					0
Female	173 (54%)	134 (38%)	34 (25%)	341 (42%)	
Male	149 (46%)	217 (62%)	102 (75%)	468 (58%)	
Age (years), median (IQR)	60 [39; 67]	68 [60; 76]	72 [64; 79]	65 [53; 73]	0
Height (cm), median (IQR)	165 [160; 171]	166 [160; 172]	170 [163; 175]	166 [160; 172]	0.008
Weight (kg), median (IQR)	64 [56; 73]	65 [55; 73]	63 [55; 74]	64 [55; 73]	0.977
Mentation, n (%)					0.46
1	322(39.80%)	344(42.52%)	132(16.32%)	798(98.64%)	
2	0(0%)	2(0.25%)	1(0.12%)	3(0.37%)	
3	0(0%)	2(0.25%)	1(0.12%)	3(0.37%)	
4	0(0%)	1(0.12%)	1(0.12%)	2(0.25%)	
5	0(0%)	1(0.12%)	0(0%)	1(0.12%)	
6	0(0%)	0(0%)	0(0%)	0(0%)	
7	0(0%)	1(0.12%)	1(0.12%)	2(0.25%)	
8	0(0%)	0(0%)	0(0%)	0(0%)	
Vital signs					
Fever peak, median (IQR)	38 [37; 39]	38 [37; 39]	38 [38; 39]	38 [37; 39]	0.002
Temperature admission, median (IQR)	37 [36; 37]	37 [37; 38]	37 [37; 38]	37 [37; 38]	0.001
Tmax during the course, median (IQR)	37 [37; 38]	37 [37; 38]	38 [37; 39]	37 [37; 38]	0
Heart rate, median (IQR)	92 [83; 100]	92 [83; 101]	91 [84; 103]	92 [83; 101]	0.824
Respiration Rate, median (IQR)	20 [18; 20]	20 [19; 20]	20 [18; 21]	20 [18; 20]	0.043
Systolic blood pressure, median (IQR)	132 [120; 145]	134 [122; 150]	133 [118; 151]	133 [120; 149]	0.228
Diastolic blood pressure, median (IQR)	80 [71; 89]	78 [70; 87]	77 [70; 85]	78 [70; 88]	0.062
CURB-65 score, n (%)					
0	183 (57%)	113 (32%)	26 (19%)	322 (40%)	
1	116 (36%)	136 (39%)	55 (40%)	307 (38%)	
2	20 (6%)	88 (25%)	44 (32%)	152 (19%)	
3	3 (1%)	14 (4%)	10 (7%)	27 (3%)	
4	0 (0.0%)	0 (0.0%)	1 (1%)	1 (0%)	
5	0 (0.0%)	0 (0.0%)	0 (0.0%)	0 (0.0%)	
PSI score, median (IQR)	55 [38; 69]	74 [60; 94]	93 [75; 112]	69 [52; 90]	0
Chief Complaint					
Cough, n (%)					0.009
No	63 (20%)	93 (26%)	44 (32%)	200 (25%)	
Yes	259 (80%)	258 (74%)	92 (68%)	609 (75%)	
Sputum, n (%)					0.117
No	144 (45%)	183 (52%)	71 (52%)	398 (49%)	
Yes	178 (55%)	168 (48%)	65 (48%)	411 (51%)	
Chest Pain, n (%)					0.225
No	296 (92%)	328 (93%)	131 (96%)	755 (93%)	
Yes	26 (8%)	23 (7%)	5 (4%)	54 (7%)	
Dyspnea, n (%)					0
No	271 (84%)	259 (74%)	87 (64%)	617 (76%)	
Yes	51 (16%)	92 (26%)	49 (36%)	192 (24%)	
Chest Tightness, n (%)					0.233
No	288 (89%)	310 (88%)	114 (84%)	712 (88%)	
Yes	34 (11%)	41 (12%)	22 (16%)	97 (12%)	
Fever, n (%)					0.215
No	158 (49%)	149 (42%)	60 (44%)	367 (45%)	
Yes	164 (51%)	202 (58%)	76 (56%)	442 (55%)	
Fatigue, n (%)					0.024
No	314 (98%)	336 (96%)	125 (92%)	775 (96%)	
Yes	8 (2%)	15 (4%)	11 (8%)	34 (4%)	
Consciousness Disorder, n (%)					0.299
No	322 (100%)	348 (99%)	136 (100%)	806 (100%)	
Yes	0 (0.0%)	3 (1%)	0 (0.0%)	3 (0%)	
Difficulty Breathing, n (%)					0.001
No	319 (99%)	343 (98%)	126 (93%)	788 (97%)	
Yes	3 (1%)	8 (2%)	10 (7%)	21 (3%)	
Hemoptysis, n (%)					0.6
No	306 (95%)	339 (97%)	130 (96%)	775 (96%)	
Yes	16 (5%)	12 (3%)	6 (4%)	34 (4%)	
Comorbidity					
Respiratory system					
COPD, n (%)					0.04
No	291 (90%)	296 (84%)	114 (84%)	701 (87%)	
Yes	31 (10%)	55 (16%)	22 (16%)	108 (13%)	
Bronchiectasis, n (%)					0.546
No	302 (94%)	332 (95%)	125 (92%)	759 (94%)	
Yes	20 (6%)	19 (5%)	11 (8%)	50 (6%)	
Emphysema bullae, n (%)					0.223
No	312 (97%)	334 (95%)	127 (93%)	773 (96%)	
Yes	10 (3%)	17 (5%)	9 (7%)	36 (4%)	
Pulmonary hypertension, n (%)					0.122
No	318 (99%)	338 (96%)	133 (98%)	789 (98%)	
Yes	4 (1%)	13 (4%)	3 (2%)	20 (2%)	
Lung cancer, n (%)					0.003
No	315 (98%)	332 (95%)	123 (90%)	770 (95%)	
Yes	7 (2%)	19 (5%)	13 (10%)	39 (5%)	
PE, n (%)					0.261
No	314 (98%)	334 (95%)	130 (96%)	778 (96%)	
Yes	8 (2%)	17 (5%)	6 (4%)	31 (4%)	
Asthma, n (%)					1
No	311 (97%)	338 (96%)	131 (96%)	780 (96%)	
Yes	11 (3%)	13 (4%)	5 (4%)	29 (4%)	
OSAHS, n (%)					0.203
No	311 (97%)	342 (97%)	128 (94%)	781 (97%)	
Yes	11 (3%)	9 (3%)	8 (6%)	28 (3%)	
Bronchitis, n (%)					0.316
No	319 (99%)	343 (98%)	133 (98%)	795 (98%)	
Yes	3 (1%)	8 (2%)	3 (2%)	14 (2%)	
Cardiovascular system					
Hypertension, n (%)					0
No	223 (69%)	204 (58%)	68 (50%)	495 (61%)	
Yes	99 (31%)	147 (42%)	68 (50%)	314 (39%)	
Atrial fibrillation, n (%)					0.001
No	312 (97%)	322 (92%)	119 (88%)	753 (93%)	
Yes	10 (3%)	29 (8%)	17 (12%)	56 (7%)	
Coronary heart disease, n (%)					0.01
No	299 (93%)	303 (86%)	116 (85%)	718 (89%)	
Yes	23 (7%)	48 (14%)	20 (15%)	91 (11%)	
Arrhythmia, n (%)					0.003
No	290 (90%)	293 (83%)	107 (79%)	690 (85%)	
Yes	32 (10%)	58 (17%)	29 (21%)	119 (15%)	
Heart failure, n (%)					0
No	305 (95%)	296 (84%)	92 (68%)	693 (86%)	
Yes	17 (5%)	55 (16%)	44 (32%)	116 (14%)	
Digestive system					
Liver dysfunction, n (%)					0.013
No	290 (90%)	294 (84%)	110 (81%)	694 (86%)	
Yes	32 (10%)	57 (16%)	26 (19%)	115 (14%)	
Gastric cancer, n (%)					0.129
No	321 (100%)	348 (99%)	133 (98%)	802 (99%)	
Yes	1 (0%)	3 (1%)	3 (2%)	7 (1%)	
Colorectal cancer, n (%)					0.093
No	318 (99%)	345 (98%)	130 (96%)	793 (98%)	
Yes	4 (1%)	6 (2%)	6 (4%)	16 (2%)	
Esophageal cancer, n (%)					0.152
No	322 (100%)	347 (99%)	135 (99%)	804 (99%)	
Yes	0 (0.0%)	4 (1%)	1 (1%)	5 (1%)	
Hepatitis, n (%)					0.654
No	322 (100%)	349 (99%)	136 (100%)	807 (100%)	
Yes	0 (0.0%)	2 (1%)	0 (0.0%)	2 (0%)	
Urinary system					
Renal Insufficiency, n (%)					0
No	315 (98%)	315 (90%)	117 (86%)	747 (92%)	
Yes	7 (2%)	36 (10%)	19 (14%)	62 (8%)	
Urological tumors, n (%)					0.006
No	321 (100%)	346 (99%)	130 (96%)	797 (99%)	
Yes	1 (0%)	5 (1%)	6 (4%)	12 (1%)	
Kidney Stones, n (%)					0.578
No	311 (97%)	343 (98%)	131 (96%)	785 (97%)	
Yes	11 (3%)	8 (2%)	5 (4%)	24 (3%)	
Endocrine system					
Diabetes, n (%)					0
No	277 (86%)	271 (77%)	97 (71%)	645 (80%)	
Yes	45 (14%)	80 (23%)	39 (29%)	164 (20%)	
Nervous system					
Senile dementia, n (%)					0.122
No	322 (100%)	348 (99%)	134 (99%)	804 (99%)	
Yes	0 (0.0%)	3 (1%)	2 (1%)	5 (1%)	
Cerebral infarction, n (%)					0
No	306 (95%)	319 (91%)	114 (84%)	739 (91%)	
Yes	16 (5%)	32 (9%)	22 (16%)	70 (9%)	
PD, n (%)					0.023
No	322 (100%)	344 (98%)	134 (99%)	800 (99%)	
Yes	0 (0.0%)	7 (2%)	2 (1%)	9 (1%)	
History of mlignancy, n (%)					0
No	307 (95%)	312 (89%)	114 (84%)	733 (91%)	
Yes	15 (4%)	39 (11%)	22 (16%)	76 (9%)	
Full Blood Count					
Hemoglobin (g/L), Mean ± SD	128 ± 15	123 ± 18	115 ± 21	124 ± 18	0
Neutrophil percent (%), median (IQR)	60 [52; 67]	75 [65; 83]	81 [70; 88]	69 [59; 81]	0
Neutrophil count (*10^9/L), median (IQR)	4 [3; 5]	5 [3; 7]	7 [4; 10]	4 [3; 7]	0
MCV (fl), median (IQR)	90 [87; 94]	91 [89; 94]	91 [89; 95]	91 [88; 94]	0.002
MCH (pg), median (IQR)	30 [29; 31]	30 [29; 32]	31 [29; 32]	30 [29; 31]	0.022
MCHC (g/L), median (IQR)	332 [326; 339]	331 [325; 341]	333 [325; 341]	332 [325; 340]	0.786
MPV (fl), median (IQR)	9 [9; 10]	9 [9; 10]	9 [8; 10]	9 [9; 10]	0.189
WBC Count (*10^9/L), median (IQR)	6 [5; 8]	6 [5; 9]	8 [6; 12]	7 [5; 9]	0
Lymphocyte percent (%), median (IQR)	30 [22; 37]	16 [10; 23]	10 [6; 17]	20 [11; 30]	0
Lymphocyte count (*10^9/L), median (IQR)	2 [2; 2]	1 [1; 1]	1 [1; 1]	1 [1; 2]	0
Eosinophil percent (%), median (IQR)	2 [1; 4]	1 [0; 2]	1 [0; 2]	2 [0; 3]	0
Monocyte percent (%), median (IQR)	7 [6; 8]	7 [5; 9]	6 [4; 9]	7 [5; 8]	0.141
Monocyte count (*10^9/L), median (IQR)	0 [0; 1]	0 [0; 1]	0 [0; 1]	0 [0; 1]	0.194
RDW (%), median (IQR)	13 [12; 13]	13 [12; 14]	14 [13; 14]	13 [12; 14]	0
Arterial Blood Gas					
HCO3 (mmol/L), median (IQR)	25 [24; 27]	25 [23; 28]	25 [21; 27]	25 [23; 27]	0.03
HCO3std (mmol/L), median (IQR)	25 [24; 26]	26 [24; 27]	25 [22; 27]	25 [24; 27]	0.223
pCO2 (kPa), median (IQR)	5 [5; 6]	5 [5; 6]	5 [4; 5]	5 [5; 6]	0
pH, median (IQR)	7 [7; 7]	7 [7; 7]	7 [7; 7]	7 [7; 7]	0
pO2 (kPa), median (IQR)	12 [11; 14]	12 [11; 15]	12 [9; 15]	12 [10; 15]	0.234
TCO2 (mmol/L), median (IQR)	50 [46; 54]	50 [45; 55]	48 [41; 55]	50 [45; 54]	0.134
Glu	5 [4; 6]	6 [5; 7]	6 [5; 8]	5 [5; 7]	0
Renal Function					
Cr (umol/L), median (IQR)	57 [49; 69]	61 [52; 74]	65 [56; 88]	60 [51; 74]	0
BUN (mmol/L), median (IQR)	5 [4; 6]	5 [4; 7]	7 [5; 10]	5 [4; 7]	0
GFR (mL/min per1.75m^2), median (IQR)	108 [93; 127]	103 [81; 122]	96 [74; 121]	104 [84; 124]	0
UA (umol/L), median (IQR)	280 [226; 354]	262 [200; 329]	270 [184; 350]	269 [210; 345]	0.007
ACE (U/L), median (IQR)	24 [18; 31]	22 [17; 30]	24 [17; 30]	24 [18; 30]	0.237
Blood lipids					
LDL-C (mmol/L), median (IQR)	3 [2; 3]	2 [2; 3]	2 [1; 2]	2 [2; 3]	0
TG (mmol/L), median (IQR)	1 [1; 2]	1 [1; 1]	1 [1; 1]	1 [1; 1]	0.018
ApoE (mg/dL), median (IQR)	4 [3; 5]	3 [3; 5]	4 [3; 5]	4 [3; 5]	0.436
Coagulation					
D-Dimer (mg/L), median (IQR)	0 [0; 1]	1 [0; 1]	1 [1; 2]	1 [0; 1]	0
TT (s), median (IQR)	14 [13; 15]	14 [13; 15]	14 [13; 15]	14 [13; 15]	0.014
APTT (s), median (IQR)	12 [11; 12]	12 [11; 13]	13 [12; 14]	12 [11; 13]	0
INR, median (IQR)	1 [1; 1]	1 [1; 1]	1 [1; 1]	1 [1; 1]	0
PTT (s), median (IQR)	31 [29; 34]	31 [28; 33]	31 [28; 34]	31 [29; 34]	0.102
ATA (%), median (IQR)	89 [79; 97]	81 [71; 90]	73 [62; 85]	83 [72; 93]	0
Inflammatory Cytokine					
IL-10 (pg/mL), median (IQR)	5 [5; 5]	5 [5; 5]	5 [5; 7]	5 [5; 5]	0
IL-1B (pg/mL), median (IQR)	5 [5; 7]	5 [5; 10]	7 [5; 14]	5 [5; 10]	0
IL-2R (U/mL), median (IQR)	440 [327; 583]	606 [465; 808]	1468 [1290;1964]	582 [412; 899]	0
IL-6 (pg/mL), median (IQR)	5 [3; 10]	9 [4; 22]	20 [8; 51]	7 [3; 19]	0
IL-8 (pg/mL), median (IQR)	23 [12; 67]	25 [15; 53]	39 [21; 77]	26 [14; 61]	0
TNF-α (pg/mL), median (IQR)	12 [8; 28]	14 [9; 28]	19 [12; 30]	15 [9; 28]	0.001
IgG4 (g/L), median (IQR)	1 [0; 1]	0 [0; 1]	1 [0; 1]	0 [0; 1]	0.188
Electrolytes					
P (mmol/L), median (IQR)	1 [1; 1]	1 [1; 1]	1 [1; 1]	1 [1; 1]	0
Cl (mmol/L), median (IQR)	105 [104; 107]	104 [102; 107]	103 [100; 106]	105 [102; 107]	0
Mg (mmol/L), median (IQR)	1 [1; 1]	1 [1; 1]	1 [1; 1]	1 [1; 1]	0.014
Potassium (mmol/L), median (IQR)	4 [4; 4]	4 [4; 4]	4 [4; 4]	4 [4; 4]	0.184
Ca (mmol/L), median (IQR)	2 [2; 2]	2 [2; 2]	2 [2; 2]	2 [2; 2]	0
Sodium (mmol/L), median (IQR)	140 [138; 142]	139 [136; 141]	136 [131; 140]	139 [136; 141]	0
Inflammation Measurements					
CRP (mg/L), median (IQR)	8 [2; 30]	34 [7; 80]	107 [37; 160]	25 [4; 78]	0
PCT(ng/mL), median (IQR)	0 [0; 0]	0 [0; 0]	0 [0; 1]	0 [0; 0]	0
ESR (mm/h), median (IQR)	32 [16; 53]	45 [24; 71]	64 [35; 90]	41 [21; 69]	0
Myocardial Enzyme					
CK-MB (U/L), median (IQR)	4 [3; 6]	5 [4; 8]	6 [4; 9]	5 [3; 7]	0
cTnI (ng/ml), median (IQR)	0 [0; 0]	0 [0; 0]	0 [0; 0]	0 [0; 0]	0
CK (U/L), median (IQR)	63 [42; 94]	67 [41; 114]	60 [34; 122]	65 [41; 103]	0.329
α-HBDH (U/L), median (IQR)	124 [104; 153]	150 [120; 182]	166 [128; 233]	142 [113; 173]	0
LDH (U/L), median (IQR)	190 [160; 220]	220 [188; 266]	268 [203; 336]	211 [176; 264]	0
Cell immunity					
CD3T (%), median (IQR)	75 [69; 80]	67 [59; 74]	69 [59; 76]	71 [64; 77]	0
CD4T (%), median (IQR)	45 [40; 50]	40 [33; 46]	40 [31; 47]	42 [35; 48]	0
CD8T (%), median (IQR)	25 [20; 31]	23 [17; 30]	24 [17; 32]	24 [18; 31]	0.023
CD3T (Cells/uL), median (IQR)	1360 [1157;1630]	687 [482; 902]	494 [343; 826]	932 [578;1284]	0
CD4T (Cells/uL), median (IQR)	827 [687;1013]	414 [267; 519]	293 [205; 480]	537 [332; 776]	0
CD64 infection index	1 [0; 1]	1 [0; 2]	2 [1; 5]	1 [0; 2]	0
CD8T (Cells/uL), median (IQR)	462 [369; 604]	226 [152; 324]	183 [100; 301]	314 [186; 460]	0
Humoral Immunity					
Ig A (g/L), median (IQR)	2 [2; 3]	3 [2; 3]	2 [2; 4]	2 [2; 3]	0.869
Ig E (IU/mL), median (IQR)	49 [18; 186]	57 [21; 162]	137 [29; 791]	58 [21; 211]	0
Ig G (g/L), median (IQR)	12 [11; 14]	12 [10; 15]	12 [10; 15]	12 [11; 15]	0.275
Ig M (g/L), median (IQR)	1 [1; 1]	1 [1; 1]	1 [0; 1]	1 [1; 1]	0
Liver Function					
GGT (U/L), median (IQR)	26 [16; 46]	30 [18; 58]	37 [24; 68]	29 [18; 55]	0
Alb (g/L), Mean ± SD	38 ± 4	35 ± 5	30 ± 5	35 ± 5	0
AST (U/L), median (IQR)	18 [12; 31]	21 [13; 34]	24 [15; 40]	20 [12; 34]	0.003
ALT (U/L), median (IQR)	18 [15; 26]	22 [17; 32]	27 [20; 46]	21 [16; 31]	0
ALP (U/L), median (IQR)	73 [59; 90]	74 [62; 92]	88 [68; 124]	75 [61; 94]	0
PA (mg/L), median (IQR)	192 [148; 230]	142 [103; 193]	98 [70; 142]	159 [109; 207]	0
TB (umol/L), median (IQR)	8 [6; 11]	9 [7; 11]	9 [7; 14]	9 [6; 12]	0.047
TP (g/L), median (IQR)	66 [63; 70]	62 [58; 67]	61 [55; 65]	64 [60; 68]	0
FIB (g/L), median (IQR)	4 [3; 5]	4 [3; 5]	5 [4; 5]	4 [3; 5]	0
Complement system					
C3 (g/L), median (IQR)	1 [1; 1]	1 [1; 1]	1 [1; 1]	1 [1; 1]	0.032
C4 (g/L), median (IQR)	0 [0; 0]	0 [0; 0]	0 [0; 0]	0 [0; 0]	0.622
CH50 (U/mL), Mean ± SD	51 ± 14	49 ± 15	48 ± 17	50 ± 15	0.11
Respiratory support					
HFNC, n (%)					0
No	319 (99%)	338 (96%)	118 (87%)	775 (96%)	
Yes	3 (1%)	13 (4%)	18 (13%)	34 (4%)	
NIMV, n (%)					0
No	317 (98%)	311 (89%)	113 (83%)	741 (92%)	
Yes	5 (2%)	40 (11%)	23 (17%)	68 (8%)	
IMV, n (%)					0.485
No	321 (100%)	347 (99%)	135 (99%)	803 (99%)	
Yes	1 (0%)	4 (1%)	1 (1%)	6 (1%)	
Clinical Outcomes					
ICU duration (days), median (IQR)	0 [0; 0]	0 [0; 6]	0 [0; 17]	0 [0; 0]	0
Ventilation duration (days), median (IQR)	0 [0; 0]	0 [0; 0]	0 [0; 4]	0 [0; 0]	0
Length of stay, median (IQR)	8 [7; 10]	10 [8; 15]	14 [9; 20]	9 [7; 14]	0
Inpatient Outcome, n (%)					0
Alive	322 (100%)	332 (95%)	115 (85%)	769 (95%)	
Dead	0 (0.0%)	19 (5%)	21 (15%)	40 (5%)	
ICU free days (days), median (IQR)	7 [6; 9]	8 [1; 11]	5 [0; 11]	7 [4; 10]	0.005
Outcome at 28 days, n (%)					0
Alive	322 (100%)	338 (96%)	119 (88%)	779 (96%)	
Dead	0 (0.0%)	13 (4%)	17 (12%)	30 (4%)	
Days from symptom onset (days), median (IQR)	10 [7; 14]	9 [5; 14]	10 [6; 14]	10 [6; 14]	0.199
ICU admission, n (%)					0
No	300 (93%)	261 (74%)	76 (56%)	637 (79%)	
Yes	22 (7%)	90 (26%)	60 (44%)	172 (21%)	

COPD, Chronic obstructive pulmonary disease; OSAHS, Obstructive sleep apnea hypopnea syndrome; MCV, Mean corpuscular volume; MCH, Mean corpuscular hemoglobin; MCHC, Mean corpuscular hemoglobin concentration; WBC, White bold cell; MPV, Mean platelet volume; RDW, Red blood cell distribution width; HCO3, Carbonic acid hydrogen radical; HCO3std, Standard bicarbonate; pCO2, Partial pressure of carbon dioxide; Ph, Potential of hydrogen; pO2, Partial pressure of oxygen; TCO2, Total carbon dioxide; Glu, Glucose; Cr, Creatinine; BUN, Blood urea nitrogen; GFR, Glomerular Filtration Rate; UA, Urine Acid; ACE, Angiotensin-Converting Enzyme; LDL-C, Low-Density Lipoprotein Cholesterol; TG, Triglyceride; ApoE, Apolipoprotein E, TT, Thrombin time; APTT, Activated partial thromboplastin time; INR, International normalized ratio; PTT, Partial thromboplastin time; ATA, Antithrombin Activity; IL-10, Lnterleukin-10; IL-1B, Lnterleukin-1B; IL-2R, Lnterleukin-2 Receptor; IL-6, Lnterleukin-6; IL-8, Lnterleukin-8; TNF-α, Tumor necrosis factor-alpha; IgG4, Immunoglobulin G4; CK-MB, Creatine kinase MB; cTnI, Cardiac troponin I; CK, Creatine kinase; α-HBDH, Alpha-hydroxybutyric dehydroge; LDH, Lactate dehydrogenase; Ig A, Immunoglobulin A; Ig E, Immunoglobulin E; Ig G, Immunoglobulin G; Ig M, Immunoglobulin M; GGT, γ-Glutamyl transferase GGT; Alb, Albumin; AST, Aspartate aminotransferase; ALT, Alanine aminotransferase; ALP, Alkaline phosphatase; PA, Prealbumin; TB, Total bilirubin; TP, Total Protein; FIB, Fibrinogen; C3, Complement C3; C4, Complement C4; CH50, 50% Hemolytic unit of Complement; HFNC, High Flow Nasal Cannula; NIMV, Noninvasive Mechanical Ventilation; IMV, Invasive Mechanical Ventilation.

### Relationship between distinct clinical immune phenotypes and clinical outcomes

**Figure 4:**
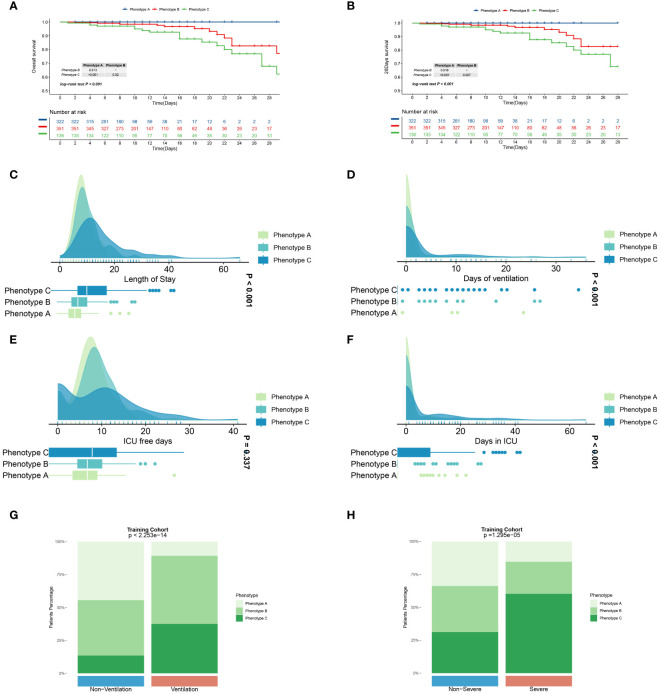
Primary and secondary outcomes among three distinct immune phenotypes in training cohort. **(A)** Survival curves for various phenotype patients during their hospitalization. **(B)** Survival curves for various phenotype patients over 28 days. Blue line represents Phenotype A patients, red for Phenotype B patients, and green for Phenotype C patients. CAP patients in Phenotype A had a better prognosis than those in Phenotype A and C (P<0.05).Phenotype C CAP patients experience extended hospital stays **(C)** and ICU stays **(F)**, prolonged ventilation days **(D)**, and fewer ICU-free days **(E)** in comparison to patients with the other two phenotypes. Green represents Phenotype A patients, light blue for Phenotype B patients, and dark blue for Phenotype C patients. Patients with phenotype C comprise a greater proportion of patients requiring assisted ventilation **(G)** and those with severe pneumonia **(H)**. Differences are observed in patient composition with respect to ventilation and the presence of severe pneumonia. P<0.001.

In current research, distinct immune phenotypes were correlated with primary and secondary outcomes. Within the training cohort, Phenotype C had the highest inpatient mortality rate, with 21 deaths (15.4%, n=136), markedly higher than that observed in Phenotype A (0 deaths, 0%, n=322) and Phenotype B (19 deaths, 5.4%, n=351) (P<0.001). Furthermore, Phenotype C experienced the highest 28-day mortality rate with 17 deaths (12.5%, n=136) compared to Phenotypes A (0 deaths, 0%, n=322) and B (13 deaths, 3.7%, n=351). Similar trends in survival outcomes were observed in the meta and validation cohorts, as detailed in **Supplementary Tables S1** and **S2**. Across all three cohorts, patients characterized as Phenotype C consistently exhibited a poorer prognosis compared to those classified as Phenotypes A and B (P<0.001; **Figures 4A, B**, **Supplementary Figures S5A, B**, and **S6A, B**). Furthermore, the three clinically derived immune phenotypes showed notable differences across all primary and secondary outcomes (**Figures 4C–H**, **Supplementary Figures S5C–H**, and **S6C–H**). Our investigation also explored the correspondence between the immune phenotypes identified in this study and traditional clinical categorizations such as CURB-65 and PSI. The results indicate that our immune phenotyping operates independently of these conventional classifications (**Figure 6I**, **Supplementary Figures S9A, B**), firmly establishing the utility and precision of our clustering approach. The presented evidence highlights distinct clinical outcomes among the phenotypes and underscores the significance of adopting this new classification in clinical practice, thereby demonstrating its practical relevance.

### Construction and evaluation of integrated machine learning signatures

**Figure 5:**
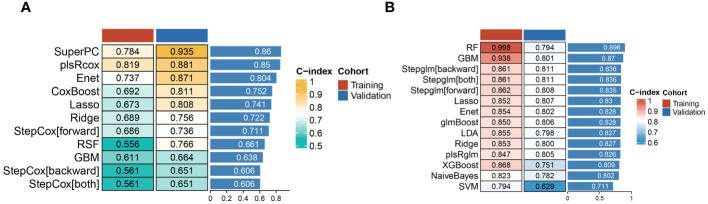
Heatmap dipicted C-index of various machine learning method in training and validation cohort for patients’ outcome **(A)** and pneumonia severity **(B)**.

**Figure 6:**
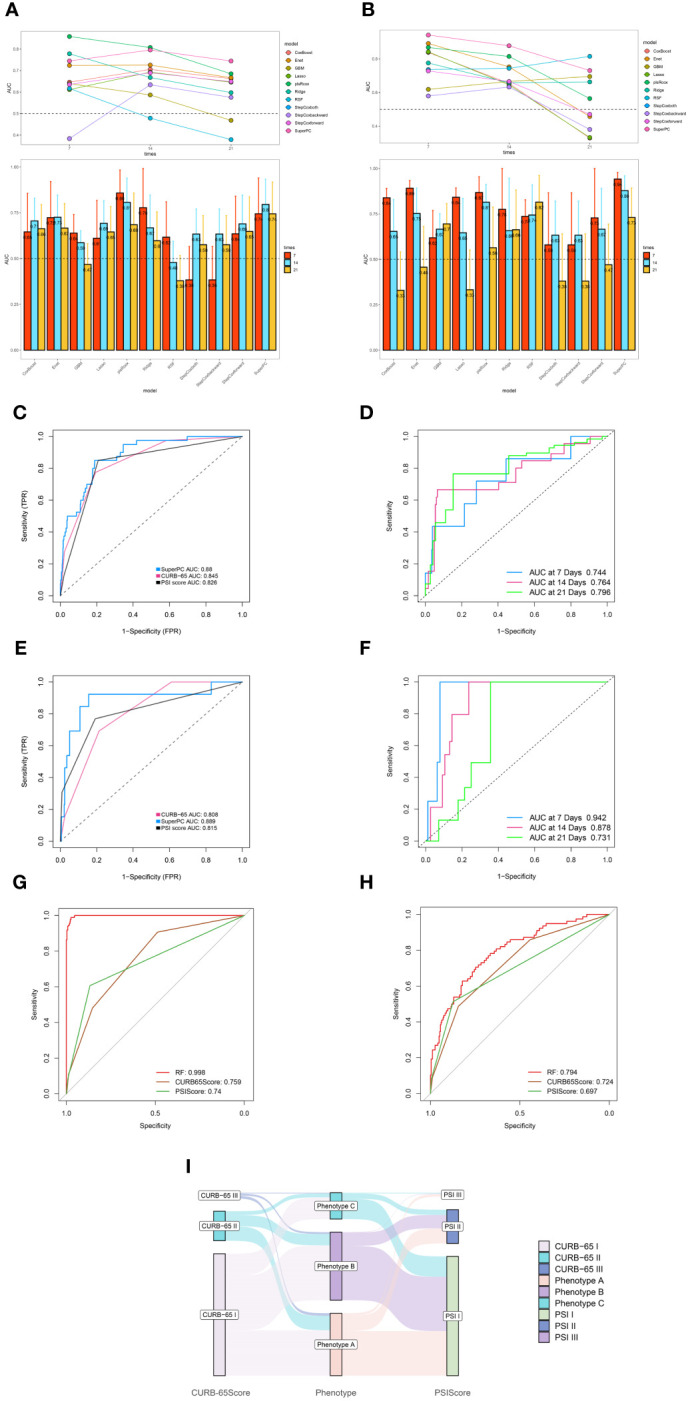
Robust performance of machine learning algorithm. **(A)** Time dependent bar and line graph of 9 machine learning methods at 7 days, 14days, and 21 days in training cohort. **(B)** Time dependent bar and line graph of 9 machine learning methods at 7 days, 14days, and 21 days in validation cohort. **(C)** The performance of SuperPC method and conventional PSI and CURB-65 evaluation criteria in training cohort. **(D)** Time dependent ROC curve of SuperPC method at 7 days, 14 days, 21 days in training cohort. **(E)** The performance of SuperPC method and conventional PSI and CURB-65 evaluation criteria in validation cohort. **(F)** Time dependent ROC curve of SuperPC method at 7 days, 14 days, 21 days in validation cohort. The performance of Random forest method and conventional PSI and CURB-65 evaluation criteria in training **(G)** and validation **(H)** cohort for predicting severe pneumonia. **(I)** Sankey plot illustrated the relationship between immune phenotypes and conventional pneumonia severity index (PSI) and CURB-65 evaluation criteria in Training cohort.

Based on the immunological laboratory indicators available at Xinhua Hospital, variables exhibiting a missing rate exceeding 20% were excluded. Consequently, 31 clinical immunological laboratory indicators were selected for model development. Contrary to previous research (7, 26), our investigation not only concentrates on the prognosis of patients with CAP but also considers the likelihood of disease severity. In recent years, machine learning has gained widespread application in medical research, demonstrating robust predictive performance (27–29). Several studies have also examined the application of machine learning in forecasting CAP outcomes (7, 30, 31). However, these investigations have predominantly utilized a narrow range of machine learning algorithms and have focused primarily on predicting mortality. Physicians should, however, consider strategies for the early identification of potentially severe pneumonia patients. To address the limitations of previous research, this study has developed survival models for patients and predictive models for assessing the severity of the risk. Nine machine learning algorithms—namely, SuperPC, PlsRocx, Elastic Net, Ridge, Lasso, stepwise Cox, Random Survival Forests (RSF), and Gradient Boosting Machine (GBM)—were applied to both training and validation cohorts to facilitate optimal model selection. The results indicated that SuperPC exhibited strong predictive performance with a training cohort C-index of 0.784 and a validation cohort C-index of 0.935, averaging at 0.86 (**Figure 5A**). Consequently, it was chosen as the superior prognostic model. The variables included in the prognostic model were presented in **Supplementary Table S3**. Additionally, in order to identify severe patients earlier, we utilized 12 common machine learning algorithms (RF, GBM, Stepglm, Lasso, Enet, Glmboost, LDA, Ridge, plsRglm, xgboost, naivebayes, and SVM) to construct a predictive model for severe pneumonia. The results indicate that the random forest algorithm demonstrated the highest predictive performance in both the training cohort and the validation cohort (training cohort C-index: 0.998, validation cohort C-index: 0.794, average C-index: 0.896, **Figure 5B**). The variables encompassed in this model are also detailed in **Supplementary Table S3**. In this study, we conducted a rigorous evaluation of ourmodels' performance through a comparative analysis with conventional evaluationmetrics by examining their Receiver Operating Characteristic (ROC) curves (see **Figures 6C–H**). Remarkably, the machine learning approaches we employed demonstrated superior performance to traditional evaluation criteria, not only within the training cohort but also in the validation cohort and meta cohort (see **Supplementary Figure S10**). This finding underscores the potential of machine learning methodologies in enhancing predictive accuracy in this context. Furthermore, we leveraged a transcriptome database related to CAP, GSE188309, which includes data from 198 patients (refer to **Supplementary Tables S4** and **S5** for details) (20, 32). Using Single Sample Gene Set Enrichment Analysis (ssGSEA), an algorithm frequently utilized for assessing immune infiltration (33), we analyzed the GSE188309 dataset and identified differences in activated CD4+ T cell levels between survivors and nonsurvivors (see **Supplementary Figure S11**). This underscores the significance of CD4+ T cells as a crucial variable in our models. Surprisingly, CD4+ T cells were incorporated into both the prognostic and predictive models, highlighting their critical role in forecasting the severity and clinical outcomes for patients with CAP. Additionally, to validate the performance of our models, we compared their Receiver Operating Characteristic (ROC) curves with those derived from conventional evaluation criteria. Collectively, our results bolster the credibility of using machine learning to predict patient prognosis.

## Discussion

In this investigation, we identified and substantiated three distinct immune phenotypes through dual clustering techniques, analyzing data from 1,165 hospitalized patients with CAP. Phenotype C emerged as indicative of a poorer prognosis, lengthier hospitalization, and an increased need for assisted ventilation. Moreover, the study employed over 20 machine-learning algorithms to forecast both the prognosis and severity of CAP.

Current literature includes descriptions of phenotypes in patients with ARDS and sepsis. In their work, Calfee et al. identified two distinct ARDS phenotypes through latent class analysis (LCA), employing biomarkers and clinical data in a retrospective examination of two randomized controlled trials (RCTs) (34). Similarly, Christopher W. Seymour and colleagues (11) delineated four sepsis phenotypes, establishing correlations between host-response patterns and clinical outcomes via retrospective analysis. The secretion of inflammatory cytokines and the modulation of immune cell activity are critical in the pathogenesis of SCAP. Consequently, assessment of the immune phenotype in individuals with CAP can enable clinicians to more accurately distinguish patients at risk of progressing to SCAP. In a study conducted by Raul M. Mackenzie (35), a cohort of 217 hospitalized CAP patients underwent evaluation of lymphocyte subsets, inflammatory mediators, and immunoglobulin subclasses, revealing a distinctive lymphopenicCAP profile. This profile, characterized by diminished CD4+ lymphocytes, elevated inflammatory responses, and reduced IgG2 concentrations, was associated with increased disease severity upon admission and a poorer overall prognosis. Notwithstanding these findings, the study was limited by a relatively small sample size and predominantly included immunocompetent patients. By contrast, our research encompasses a broader demographic and a significantly larger sample size, thus providing a more comprehensive understanding of the immunological landscape in CAP. This study aimed to delineate immune phenotypes that correlate with the prognosis of patients with CAP. Analysis of 31 immunological and inflammatory parameters was conducted through unsupervised clustering, employing the “Consensus clustering” algorithm. We identified three distinct CAP immune phenotypes: Phenotype A emerged as the least severe, characterized by the lowest deviations in laboratory markers and organ function. In stark contrast, Phenotype C represented the most critical illness phenotype, marked by an increased frequency of ICU admissions and prevalence among elderly patients. Phenotype B represented an intermediate level of severity. The early detection of Phenotype C is thus crucial for improving outcomes in SCAP patients. Intriguingly, these immunophenotypes could not be completely accounted for by traditional severity scores such as the PSI and CURB-65. Most patients with low PSI and CURB-65 scores were classified under Phenotypes A and B; however, a minority presented with the high-risk Phenotype C. Therefore, incorporating immunophenotyping into the assessment offers a valuable tool for the early recognition of high-risk patients, who score low on CURB-65 and PSI indices, significantly contributing to the enhancement of their clinical prognosis.

Although immune phenotypes offer valuable insights, they do not achieve the prognostic precision of predictive models. To assess the reliability of immune phenotypes in real-world clinical contexts, we examined the association between immune phenotype classification and clinical outcomes. Our findings indicated that the three deduced immune phenotypes exhibited significant disparities across all primary and secondary outcomes measured. Notably, patients categorized within Phenotype C experienced poorer prognostic outcomes compared to those with Phenotypes A and B. These observations underscore the utility of immune phenotype classification for prognostic evaluation in patients with CAP.

Multiple studies have demonstrated the efficacy of ML in enhancing mortality predictions for patients with CAP. Cilloniz et al. reported that an adapted SeF model employing ML exhibited promise in augmenting the accuracy of mortality predictions for CAP patients within the context of a derivation-validation retrospective study (6). Despite such advancements, research on prognostic models for CAP that incorporate immunological markers remains scarce. In our investigation, we performed an analysis of data derived from the immunological laboratory indicators of CAP patients. Our findings indicate that the prognostic model established via the SuperPC algorithm demonstrates a robust predictive capability. When juxtaposed with existing models, such as CURB-65 and PSI, our model achieves a comparable mean C-index, suggesting its utility as an adjunctive tool for the clinical assessment of CAP patients. Our research not only corroborates the existing literature regarding CAP patient prognosis but also extends the analysis to encompass the likelihood of the severity of the disease. In evaluating 12 different ML algorithms, we ascertained that the Random Forest algorithm delivers a superior mean C-index, which signifies a more potent predictive performance specifically for patients with SCAP.

Lymphocytopenia has been acknowledged as an independent risk factor for adverse outcomes in patients with CAP (36). The cause of lymphocytopenia is unknown, although several causes have been proposed, such as increased apoptosis, limitations in the host immune system’s mobilization of these cells, or compartmentation at the site of infection ADDIN EN.CITE (35). Variations in lymphocyte subsets, particularly in CD4 T cells, have been implicated in the immunopathogenesis of CAP. Indeed, alterations in CD4 T cells have been associated with increased disease severity, particularly in the elderly and frail patient populations (37, 38). Our study corroborates these findings by demonstrating the prognostic and predictive significance of CD4 T cell changes. Furthermore, an analysis of the GSE188309 dataset reinforced the observation of a discernible difference in the activation levels of CD4 T cells between survivors and non-survivors of CAP. These results underscore the pivotal role of CD4 T cells in forecasting clinical outcomes and determining disease severity in CAP patients. At present, the mechanism of CD4 T cells participating in CAP is complex and not completely clear. More studies are focused on the mechanism of CD4+T cells in pneumonia caused by viral infection, especially pneumonia caused by SARS-CoV-2. CD4+T cells can differentiate into a range of helper and effector cell types, thereby exerting antiviral capabilities. Virus-specific CD4+ T cells differentiate into Th1 cells and T follicular help-er cells (Tfh). Th1 cells have antiviral activity by producing IFNγ and related cytokines. Specific circulating Tfh cells (cTfh) are produced during acute SARS-CoV-2 infection (39). A study by Liu et al. analyzed the lymphocyte subsets of COVID-19-associated pneumonia and CAP and showed that CD16+CD56+%, CD4+/CD8+ ratio, CD19+, and CD3+CD4+ independently predicted differentiation of COVID-19 and CAP. CD3+CD4+ and CD3+CD8+ counts were independent predictors of disease severity (40).

For pneumonia caused by other pathogens, regulatory CD4+CD25+ T cells were found to suppress respiratory inflammation by promoting IL-17 and IFN-γ responses in a mouse model of mycoplasma pneumonia (41). However, the exact mechanisms underlying these observations in our study warrant comprehensive investigation through basic experimental research.

## Conclusion

Our study’s principal finding demonstrates that evaluating immunological parameters upon hospital admission assists in stratifying CAP patients into three distinct immune phenotypes. Moreover, these immune phenotypes show a strong correlation with patient prognoses. We also discerned significant predictive capabilities within the SuperPC algorithm, suggesting its utility as an ancillary tool for assessing CAP. Notably, our investigation constitutes the most extensive analysis of CAP clinical phenotypes to date. An additional strength of this study is its breadth; rather than focusing on CAP related to specific pathogens, it encompasses a comprehensive evaluation of the immunophenotypes across the spectrum of CAP. This approach allows for patient classification and tailored intervention prior to the confirmation of precise etiologic agents, offering crucial guidance, especially for cases where pathogen identification proves challenging. Moreover, the rigorous application of inclusion and exclusion criteria enhances the applicability of our findings, rendering the results of significant relevance to the broader patient population.

## Limitation

Our study possesses several limitations. Firstly, its scope is confined to a single center, which may not be representative of broader populations, in contrast to multi-center studies. Secondly, the retrospective nature of our research necessitates the implementation of a prospective study to corroborate our findings and inform future clinical practice. Looking ahead, the inclusion of a wider range of variables beyond immunological indicators will enable a more comprehensive assessment of multi-organ involvement in patients with CAP. And finally, other experiment methods for example flow mass spectrometry can be applied in figuring out the potential mechanism of CD4 T cells in CAP.

## Data availability statement

The data analyzed in this study is subject to the following licenses/restrictions: Contact corresponding author for the dataset. Requests to access these datasets should be directed guoxuejun@xinhuamed.com.cn.

## Ethics statement

The studies involving humans were approved by Institutional Review Board (IRB) of Xinhua Hospital, Shanghai Jiao Tong University School of Medicine, Shanghai, China. The studies were conducted in accordance with the local legislation and institutional requirements. Written informed consent for participation was not required from the participants or the participants’ legal guardians/next of kin in accordance with the national legislation and institutional requirements.

## Author contributions

QQ: Writing – review & editing, Writing – original draft, Visualization, Validation, Software, Resources, Project administration, Methodology, Investigation, Formal Analysis, Data curation, Conceptualization. HY: Writing – original draft, Investigation, Conceptualization. JZ: Writing – original draft, Investigation, Conceptualization. XX: Writing – review & editing, Data curation. QL: Writing – review & editing, Visualization, Conceptualization. WG: Writing – review & editing, Writing – original draft, Supervision, Software, Funding acquisition, Conceptualization. XG: Writing – review & editing, Supervision, Conceptualization.

## Supplementary material

Supplementary Table 1 Characteristics and outcomes of patients divided by immune phenotypes in validation cohort.

Supplementary Table 2 Characteristics and outcomes of patients divided by immune phenotypes in meta cohort.

Supplementary Table 3 Features incorporated in prognostic and diagnostic model.

Supplementary Table 4 Expression matrix of GSE188309.

Supplementary Table 5 Clinical information of GSE188309.

Supplementary Figure 1 Graphic flowchart of this research.

Supplementary Figure 2 Visualization of clustering and dimensionality reduction results of training cohort. **(A)** Nbclust method illustrated the optimal groups of training cohort was three. **(B)** Principal Component Analysis (PCA) method successfully divided CAP patients into three distinct immune phenotypes. **(C)** Uniform Manifold Approximation and Projection (UMAP) method successfully divided CAP patients into three distinct immune phenotypes. Patients with phenotype B are represented by a yellow dot, the blue dots represent patients belong to phenotype and those with phenotype C by a purple dot.

Supplementary Figure 3 Consensus Clustering and dimensionality reduction visualization in validation cohort. **(A)** Identification of three immune phenotypes of community acquired pneumonia (CAP) patients by consensus clustering. **(B)** Cumulative distribution function (CDF) curve illustrated consensus distribution for each phenotype. **(C)** Visualization of Nbclust method in determining optimal clusters of CAP patients. **(D)** T-distributed stochastic neighbor embedding(t-SNE) method successfully divided CAP patients into three distinct immune phenotypes. **(E)** Uniform Manifold Approximation and Projection (UMAP) method successfully divided CAP patients into three distinct immune phenotypes. **(F)** Principal Component Analysis (PCA) method successfully divided CAP patients into three distinct immune phenotypes.

Supplementary Figure 4 Consensus Clustering and dimensionality reduction visualization in meta cohort. **(A)** Identification of three immune phenotypes of community acquired pneumonia(CAP) patients by consensus clustering. **(B)** Cumulative distribution function(CDF) curve illustrated consensus distribution for each phenotype. **(C)** Visualization of Nbclust method in determining optimal clusters of CAP patients. **(D)** T-distributed stochastic neighbor embedding(t-SNE) method successfully divided CAP patients into three distinct immune phenotypes. **(E)** Uniform Manifold Approximation and Projection(UMAP) method successfully divided CAP patients into three distinct immune phenotypes. **(F)** Principal Component Analysis (PCA) method successfully divided CAP patients into three distinct immune phenotypes.

Supplementary Figure 5 Primary and secondary outcomes among three distinct immune phenotypes in validation cohort. **(A)** Survival curves for various phenotype patients during their hospitalization. **(B)** Survival curves for various phenotype patients over 28 days. Blue line represents Phenotype A patients, red for Phenotype B patients, and green for Phenotype C patients. Phenotype A had a better prognosis than those in Phenotype A and C (P<0.05).Phenotype C CAP patients experience extended hospital stays **(C)**, prolonged ventilation days **(D)**, ICU stays **(F)** and fewer ICU-free days **(E)** in comparison to patients with the other two phenotypes. Green represents Phenotype A patients, light blue for Phenotype B patients, and dark blue for Phenotype C patients. CAP patients in Patients with phenotype C comprise a greater proportion of patients requiring assisted ventilation **(G)** and those with severe pneumonia **(H)**. Differences are observed in patient composition with respect to ventilation and the presence of severe pneumonia. P<0.001.

Supplementary Figure 6 Primary and secondary outcomes among three distinct immune phenotypes in meta cohort. **(A)** Survival curves for various phenotype patients during their hospitalization. **(B)** Survival curves for various phenotype patients over 28 days. Blue line represents Phenotype A patients, red for Phenotype B patients, and green for Phenotype C patients. Phenotype A had a better prognosis than those in Phenotype A and C (P<0.05).Phenotype C CAP patients experience extended hospital stays **(C)**, prolonged ventilation days **(D)**, ICU stays **(F)** and fewer ICU-free days **(E)** in comparison to patients with the other two phenotypes. Green represents Phenotype A patients, light blue for Phenotype B patients, and dark blue for Phenotype C patients. CAP patients in Patients with phenotype C comprise a greater proportion of patients requiring assisted ventilation **(G)** and those with severe pneumonia **(H)**. Differences are observed in patient composition with respect to ventilation and the presence of severe pneumonia. P<0.001.

Supplementary Figure 7 Association and variation between clinical immunological indicators and three phenotypes. Chord diagram **(A-D)** of the association between clinical immunological variables and each phenotype in validation cohort. Different phenotypes were shown in different colors: phenotype A is purple, phenotype B is blue, and phenotype C is green. Rank plot **(E-G)** of variable mean among various phenotypes in training cohort. Variables were normalized by mean and standard error.

Supplementary Figure 8 Association and variation between clinical immunological indicators and three phenotypes. Chord diagram **(A-D)** of the association between clinical immunological variables and each phenotype in meta cohort. Different phenotypes were shown in different colors: phenotype A is purple, phenotype B is blue, and phenotype C is green. Rank plot **(E-G)** of variable mean among various phenotypes in training cohort. Variables were normalized by mean and standard error.

Supplementary Figure 9 Sankey plot illustrated the relationship between immune phenotypes and conventional pneumonia severity index (PSI) and CURB-65 evaluation criteria in validation **(A)** cohort and meta cohort **(B)**.

Supplementary Figure 10 Robust performance of machine learning algorithm. **(A)** The performance of riskscore and conventional PSI and CURB-65 evaluation criteria in meta cohort. **(B)** Time dependent ROC curve of riskscore method at 7 days, 14 days, 21 days in meta cohort. **(C)** The performance of Random forest method and conventional PSI and CURB-65 evaluation criteria in meta cohort.

Supplementary Figure 11 The immune infiltration landscape of patients with community acquired pneumonia. **(A)** Box plot illustrated different types of immune cell between alive and deceased CAP patients. **(B)** Cellular interaction of immune cell types. Positive correlation is indicated in red and negative correlation in blue.
