# Supplementary figures and images for: Machine learning-based derivation and validation of three immune phenotypes for risk stratification and prognosis in community-acquired pneumonia: a retrospective cohort study
Source: Front Immunol. 2024 Jul 24;15:1441838. doi: 10.3389/fimmu.2024.1441838 (PMC11303239; doi:10.3389/fimmu.2024.1441838)

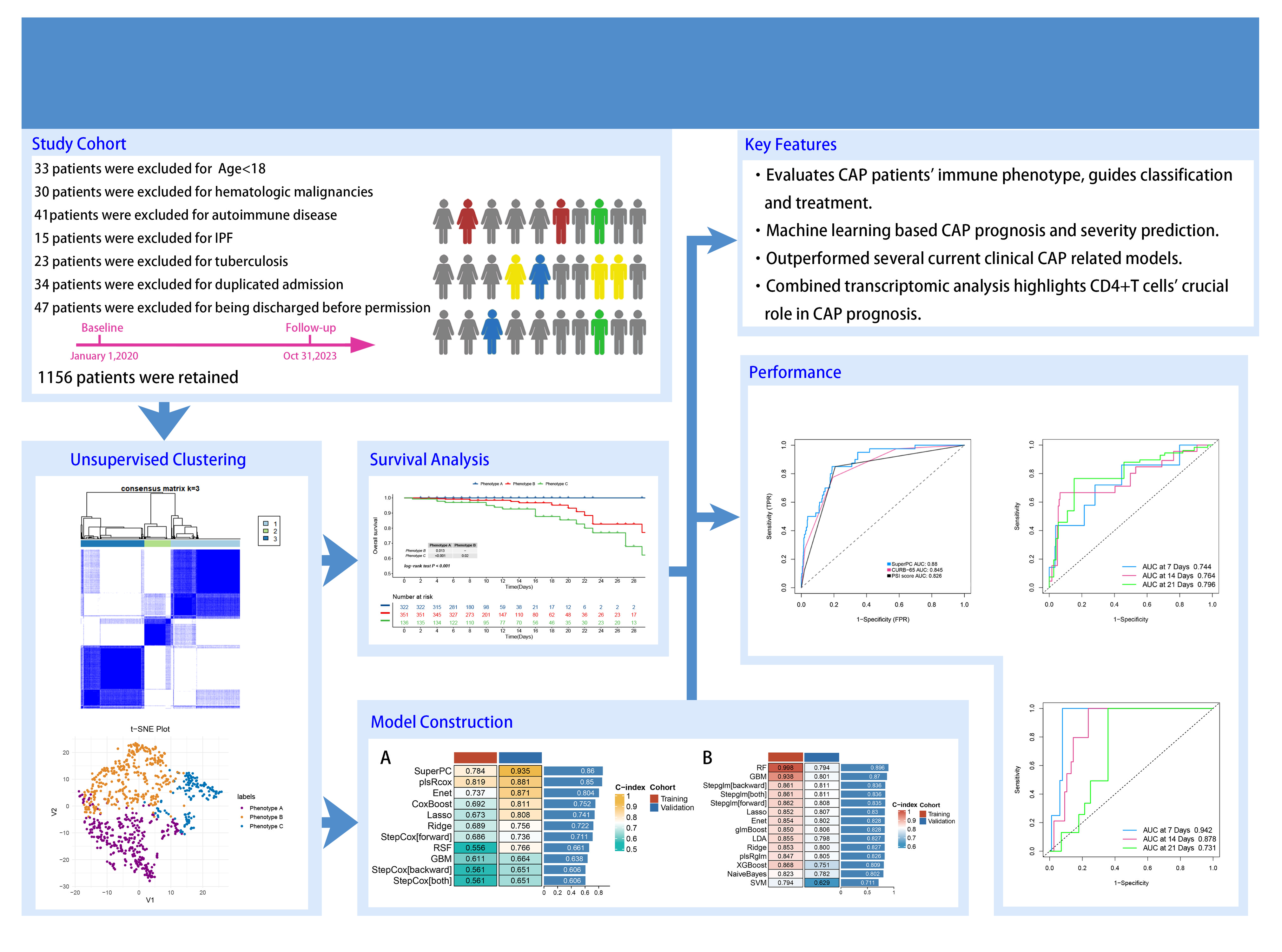

Supplement: Supplementary Figure 1 — Graphic flowchart of this research. [file Image_1.tiff]

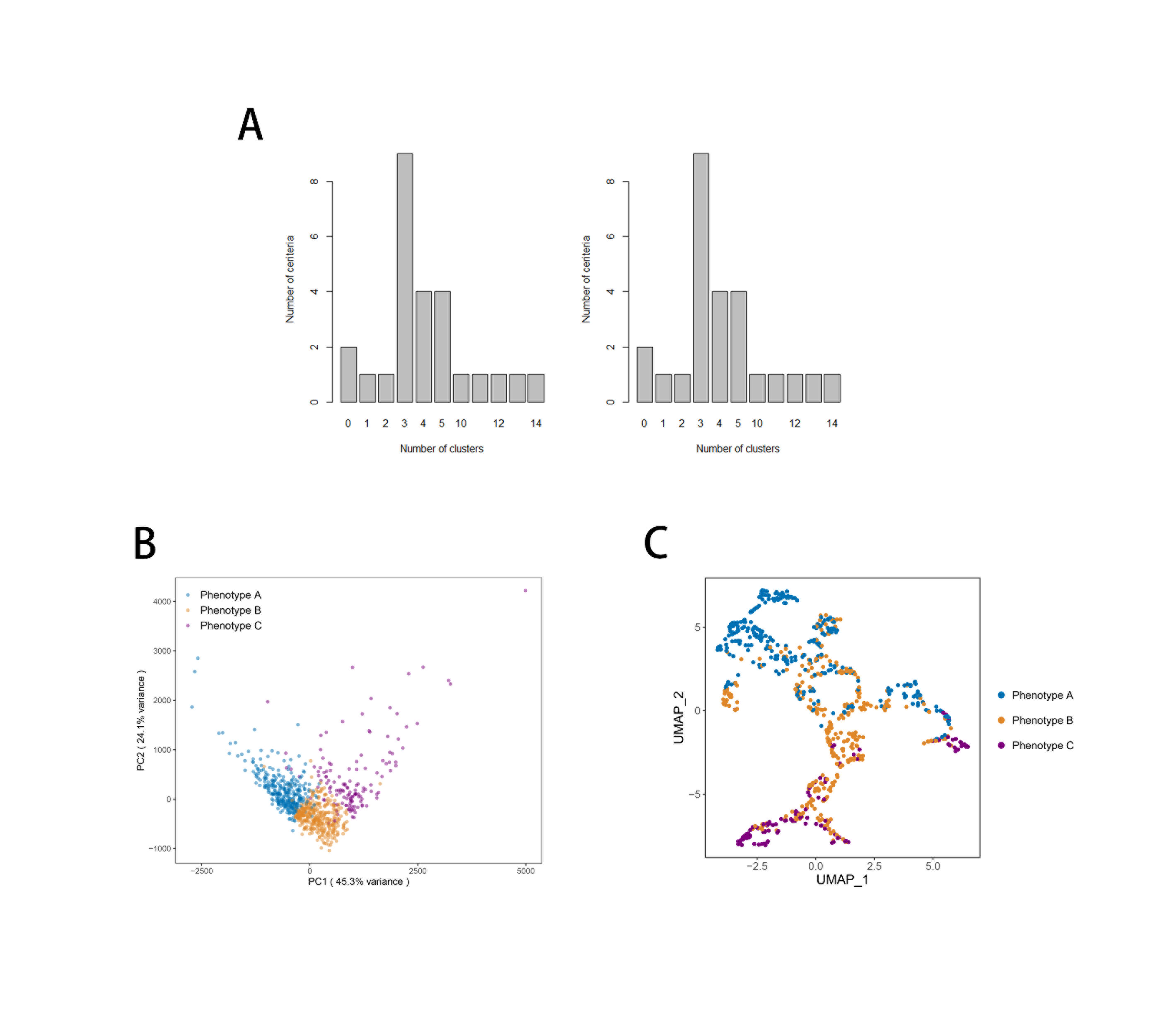

Supplement: Supplementary Figure 2 — Visualization of clustering and dimensionality reduction results of training cohort. (A) Nbclust method illustrated the optimal groups of training cohort was three. (B) Principal Component Analysis (PCA) method successfully divided CAP patients into three distinct immune phenotypes. (C) Uniform Manifold Approximation and Projection (UMAP) method successfully divided CAP patients into three distinct immune phenotypes. Patients with phenotype B are represented by a yellow dot, the blue dots represent patients belong to phenotype and those with phenotype C by a purple dot. [file Image_2.tiff]

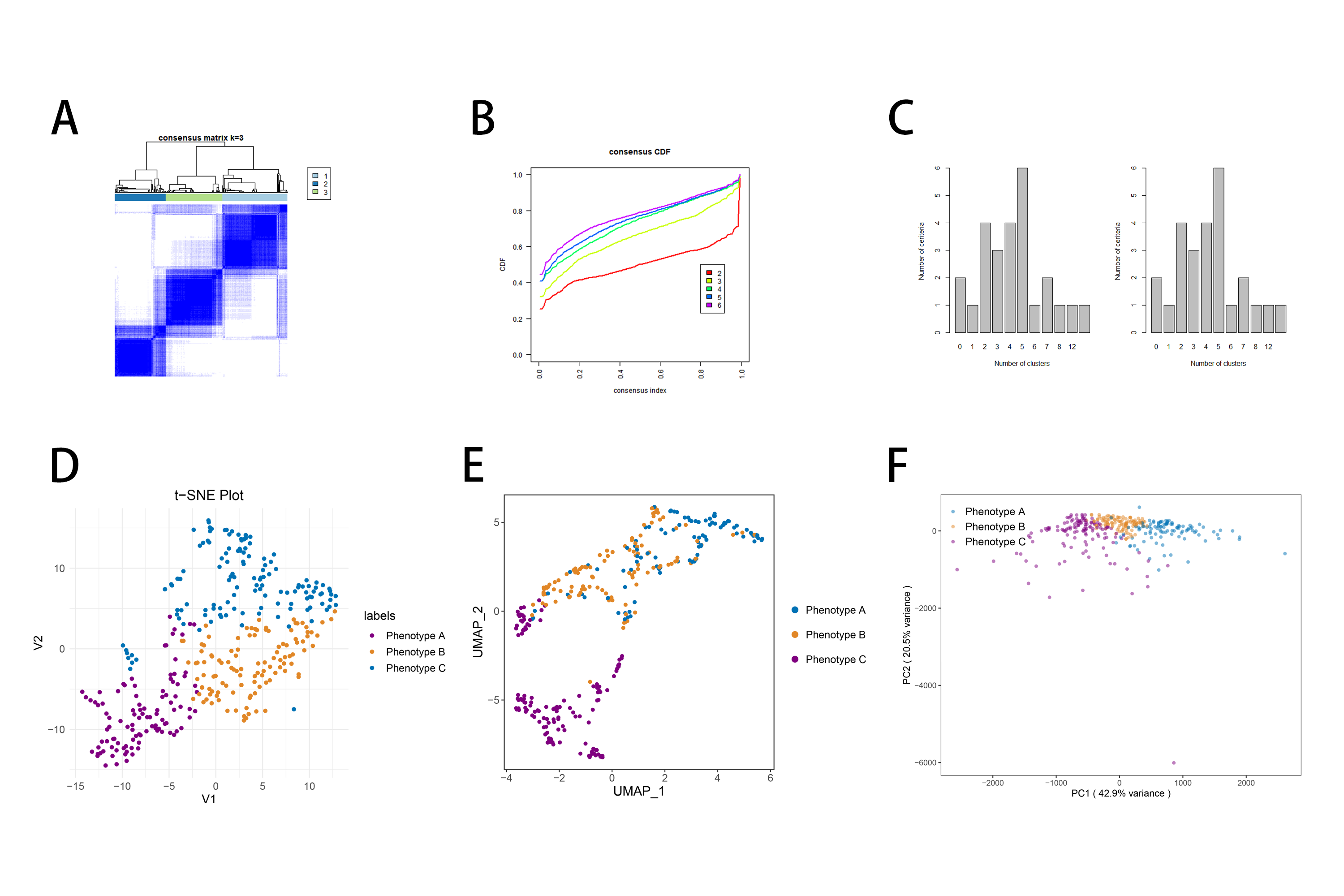

Supplement: Supplementary Figure 3 — Consensus Clustering and dimensionality reduction visualization in validation cohort. (A) Identification of three immune phenotypes of community acquired pneumonia (CAP) patients by consensus clustering. (B) Cumulative distribution function (CDF) curve illustrated consensus distribution for each phenotype. (C) Visualization of Nbclust method in determining optimal clusters of CAP patients. (D) T-distributed stochastic neighbor embedding(t-SNE) method successfully divided CAP patients into three distinct immune phenotypes. (E) Uniform Manifold Approximation and Projection (UMAP) method successfully divided CAP patients into three distinct immune phenotypes. (F) Principal Component Analysis (PCA) method successfully divided CAP patients into three distinct immune phenotypes. [file Image_3.tiff]

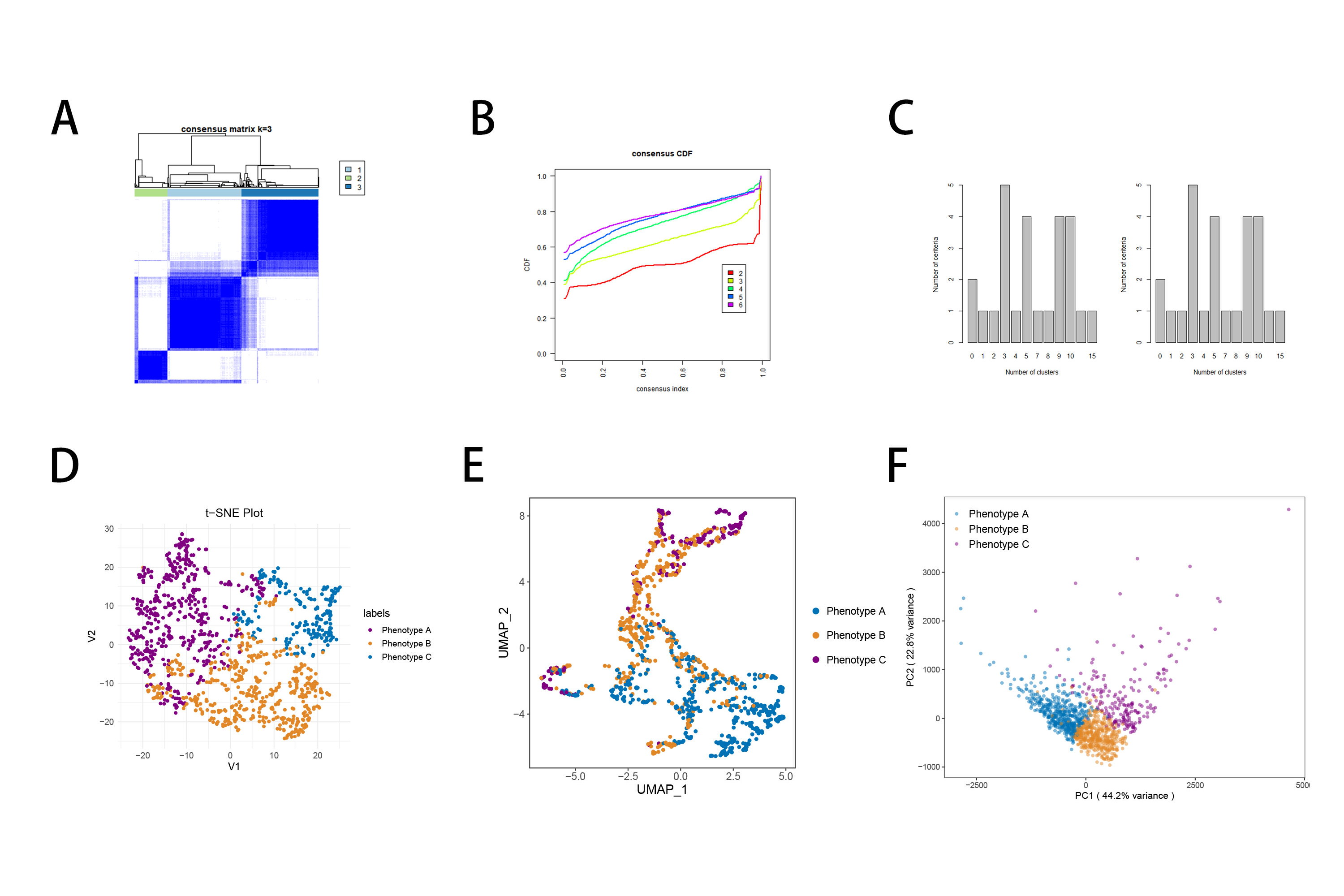

Supplement: Supplementary Figure 4 — Consensus Clustering and dimensionality reduction visualization in meta cohort. (A) Identification of three immune phenotypes of community acquired pneumonia(CAP) patients by consensus clustering. (B) Cumulative distribution function(CDF) curve illustrated consensus distribution for each phenotype. (C) Visualization of Nbclust method in determining optimal clusters of CAP patients. (D) T-distributed stochastic neighbor embedding(t-SNE) method successfully divided CAP patients into three distinct immune phenotypes. (E) Uniform Manifold Approximation and Projection(UMAP) method successfully divided CAP patients into three distinct immune phenotypes. (F) Principal Component Analysis (PCA) method successfully divided CAP patients into three distinct immune phenotypes. [file Image_4.tiff]

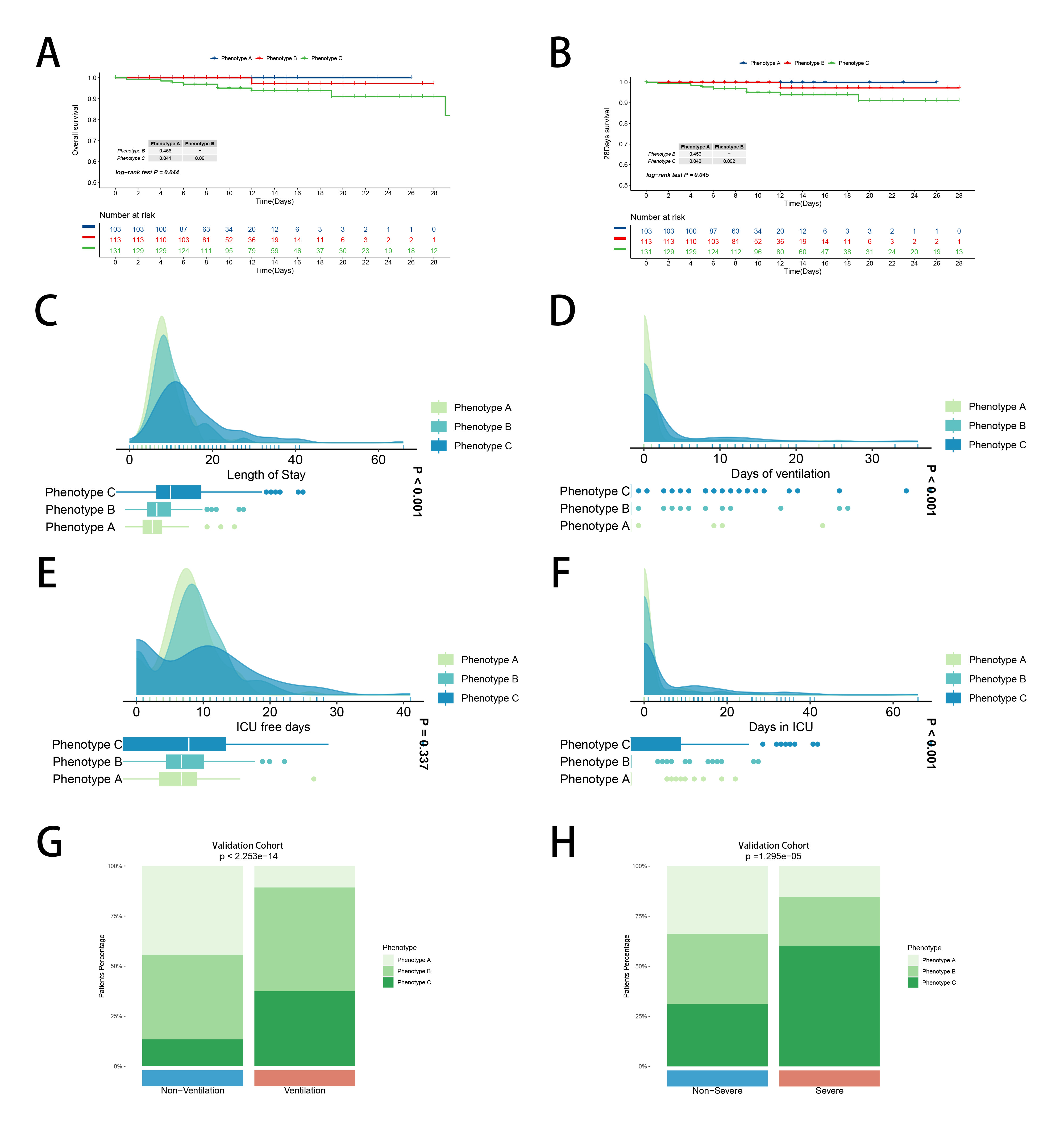

Supplement: Supplementary Figure 5 — Primary and secondary outcomes among three distinct immune phenotypes in validation cohort. (A) Survival curves for various phenotype patients during their hospitalization. (B) Survival curves for various phenotype patients over 28 days. Blue line represents Phenotype A patients, red for Phenotype B patients, and green for Phenotype C patients. Phenotype A had a better prognosis than those in Phenotype A and C (P<0.05).Phenotype C CAP patients experience extended hospital stays (C), prolonged ventilation days (D), ICU stays (F) and fewer ICU-free days (E) in comparison to patients with the other two phenotypes. Green represents Phenotype A patients, light blue for Phenotype B patients, and dark blue for Phenotype C patients. CAP patients in Patients with phenotype C comprise a greater proportion of patients requiring assisted ventilation (G) and those with severe pneumonia (H). Differences are observed in patient composition with respect to ventilation and the presence of severe pneumonia. P<0.001. [file Image_5.tiff]

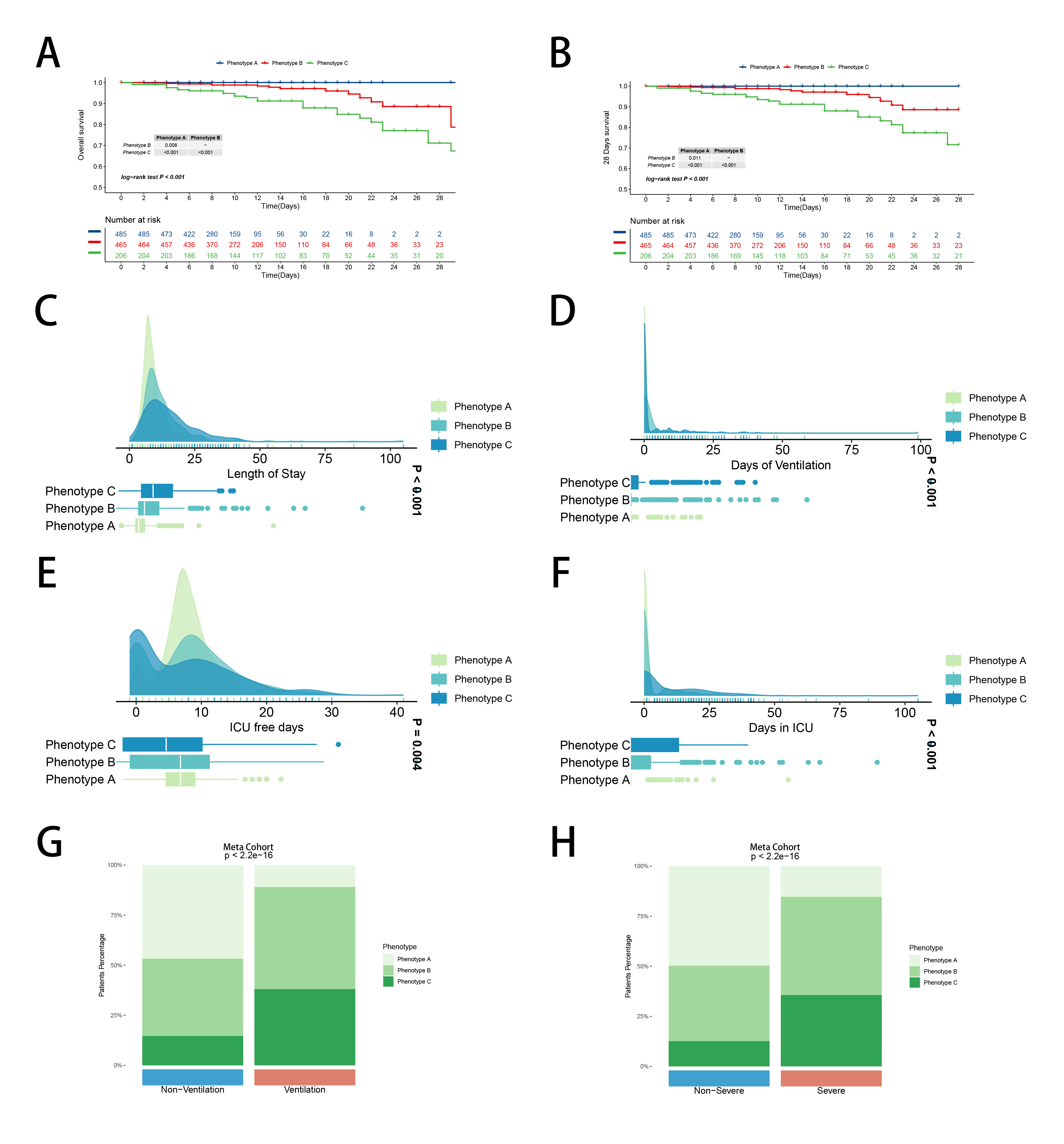

Supplement: Supplementary Figure 6 — Primary and secondary outcomes among three distinct immune phenotypes in meta cohort. (A) Survival curves for various phenotype patients during their hospitalization. (B) Survival curves for various phenotype patients over 28 days. Blue line represents Phenotype A patients, red for Phenotype B patients, and green for Phenotype C patients. Phenotype A had a better prognosis than those in Phenotype A and C (P<0.05).Phenotype C CAP patients experience extended hospital stays (C), prolonged ventilation days (D), ICU stays (F) and fewer ICU-free days (E) in comparison to patients with the other two phenotypes. Green represents Phenotype A patients, light blue for Phenotype B patients, and dark blue for Phenotype C patients. CAP patients in Patients with phenotype C comprise a greater proportion of patients requiring assisted ventilation (G) and those with severe pneumonia (H). Differences are observed in patient composition with respect to ventilation and the presence of severe pneumonia. P<0.001. [file Image_6.tiff]

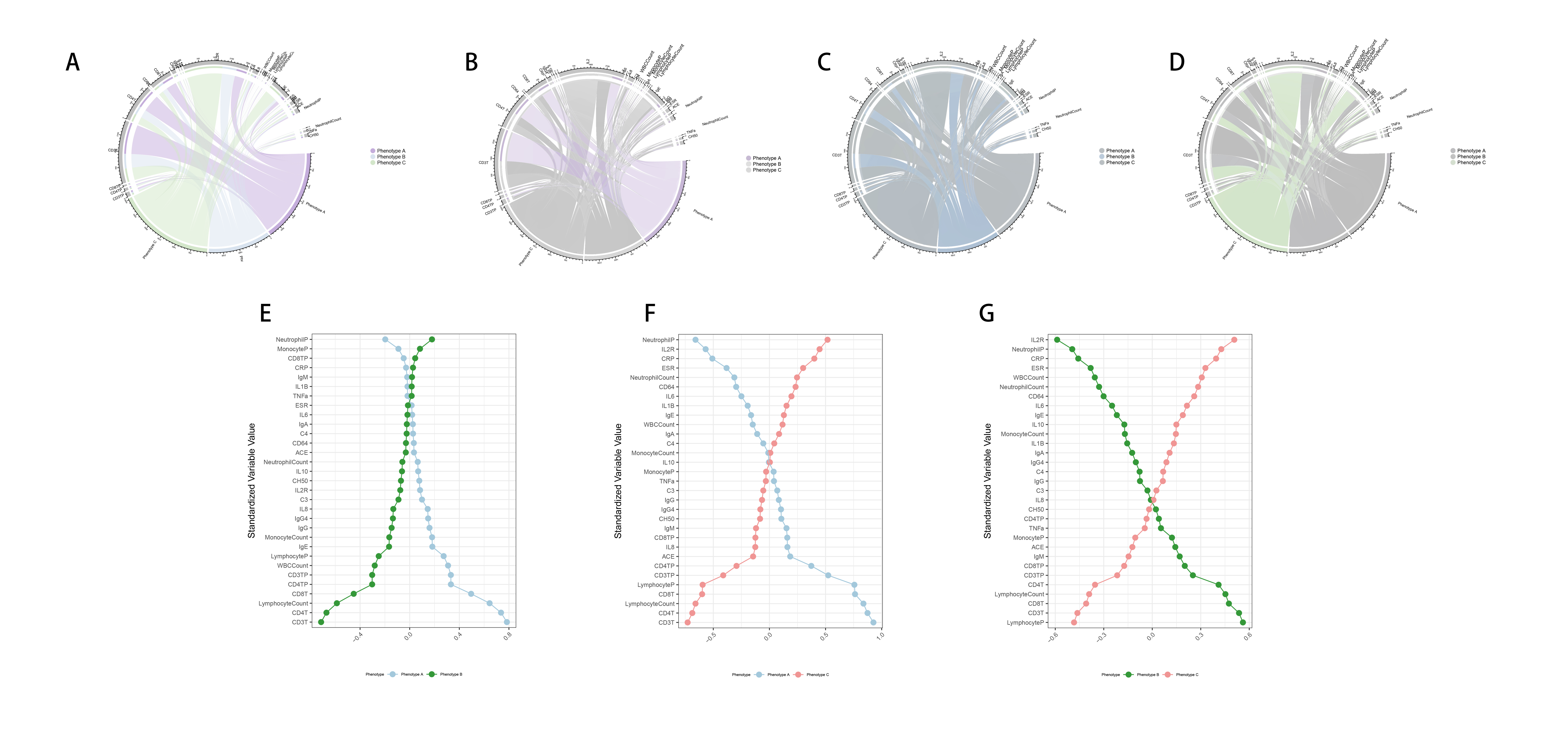

Supplement: Supplementary Figure 7 — Association and variation between clinical immunological indicators and three phenotypes. Chord diagram (A-D) of the association between clinical immunological variables and each phenotype in validation cohort. Different phenotypes were shown in different colors: phenotype A is purple, phenotype B is blue, and phenotype C is green. Rank plot (E-G) of variable mean among various phenotypes in training cohort. Variables were normalized by mean and standard error. [file Image_7.tiff]

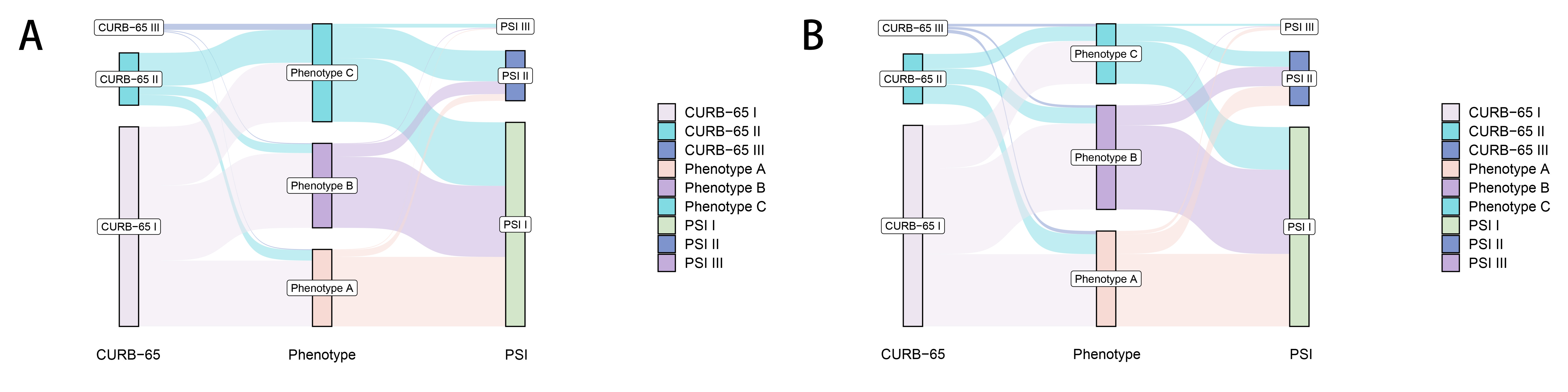

Supplement: Supplementary Figure 9 — Sankey plot illustrated the relationship between immune phenotypes and conventional pneumonia severity index (PSI) and CURB-65 evaluation criteria in validation (A) cohort and meta cohort (B). [file Image_9.tiff]

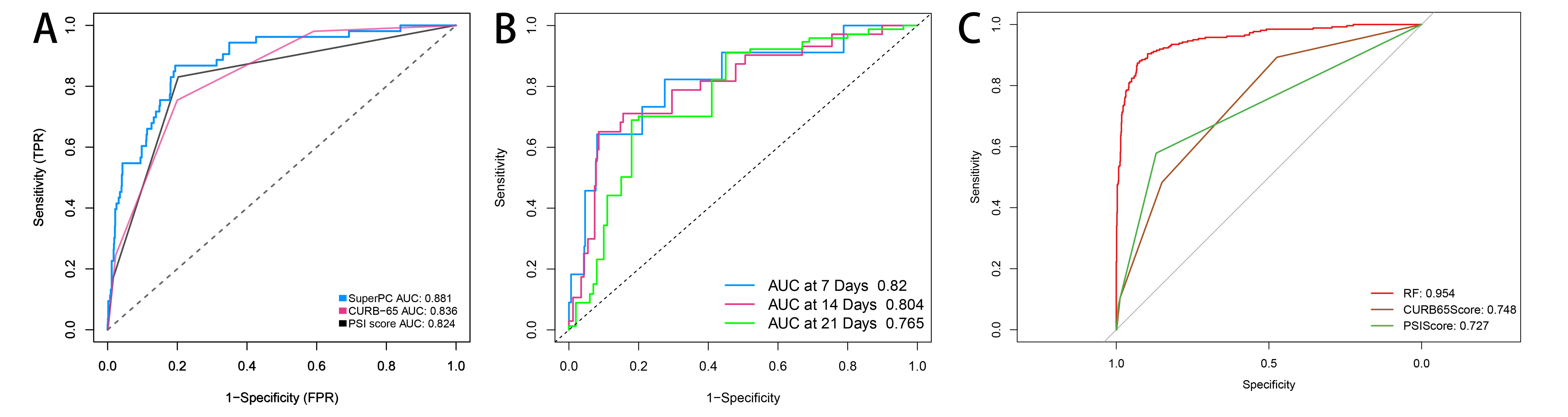

Supplement: Supplementary Figure 10 — Robust performance of machine learning algorithm. (A) The performance of riskscore and conventional PSI and CURB-65 evaluation criteria in meta cohort. (B) Time dependent ROC curve of riskscore method at 7 days, 14 days, 21 days in meta cohort. (C) The performance of Random forest method and conventional PSI and CURB-65 evaluation criteria in meta cohort. [file Image_10.tiff]

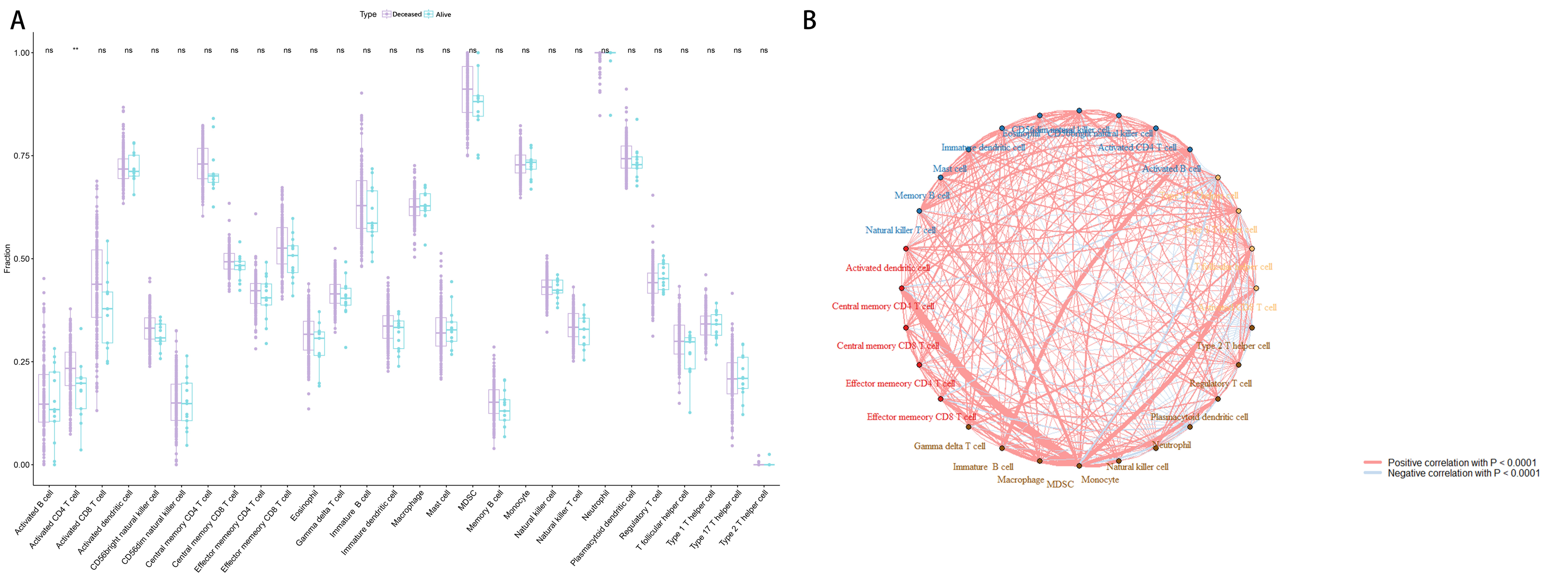

Supplement: Supplementary Figure 11 — The immune infiltration landscape of patients with community acquired pneumonia. (A) Box plot illustrated different types of immune cell between alive and deceased CAP patients. (B) Cellular interaction of immune cell types. Positive correlation is indicated in red and negative correlation in blue. [file Image_11.jpg]

# ROC Plot

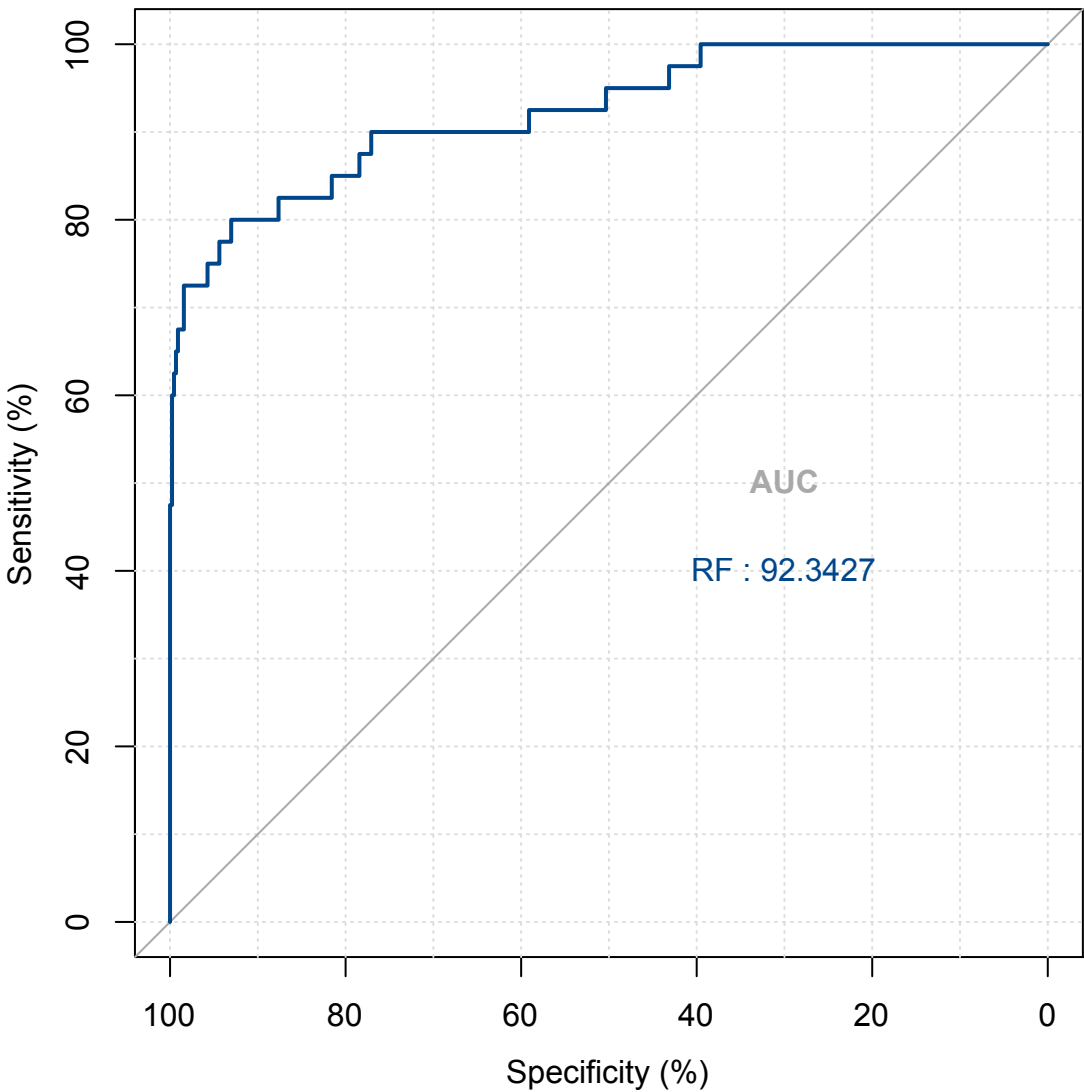

Supplement: Supplementary file 17 [file DataSheet_3.zip › Predictive model/meta-cohort-Phenotype A.pdf]

# ROC Plot

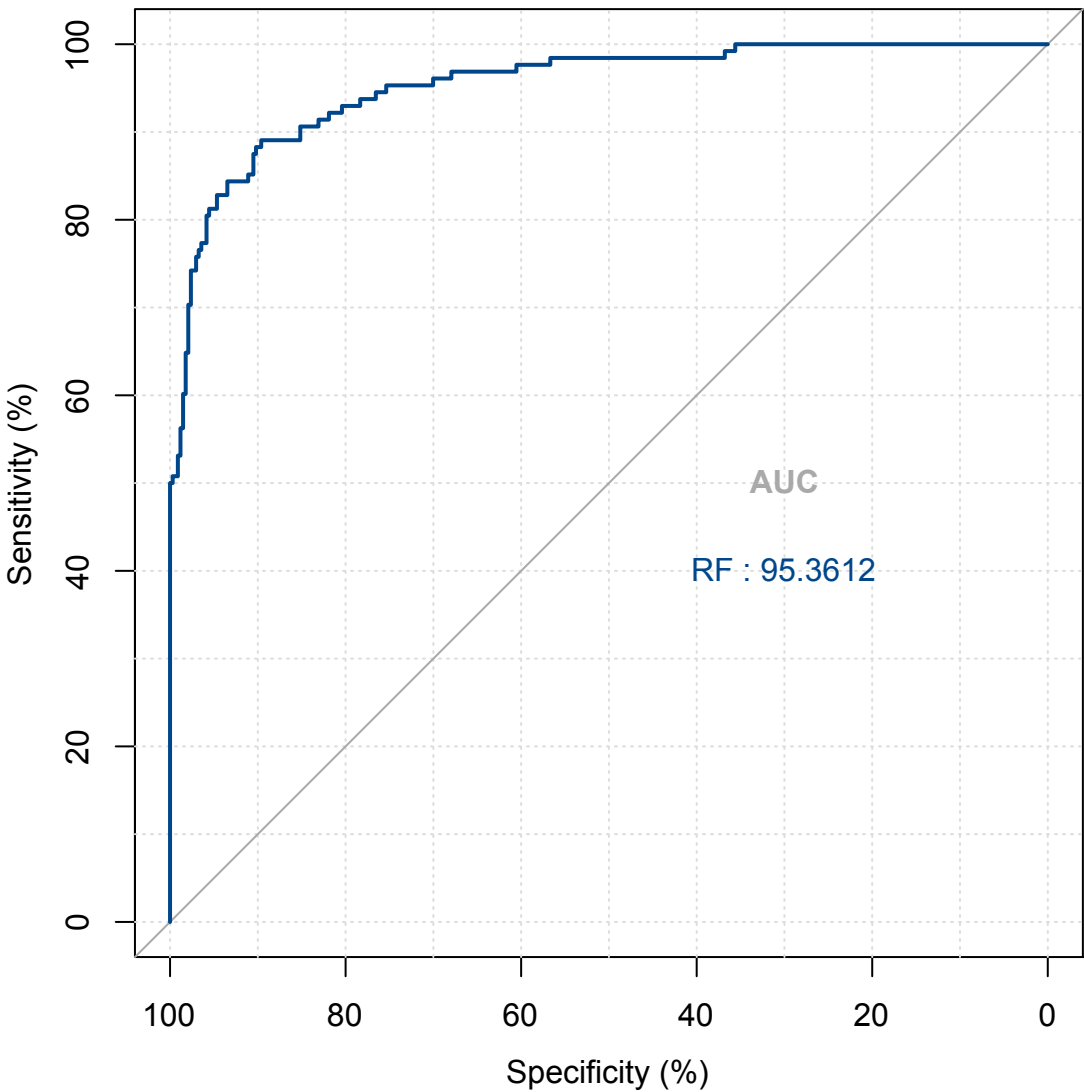

Supplement: Supplementary file 17 [file DataSheet_3.zip › Predictive model/meta-cohort-Phenotype B.pdf]

# ROC Plot

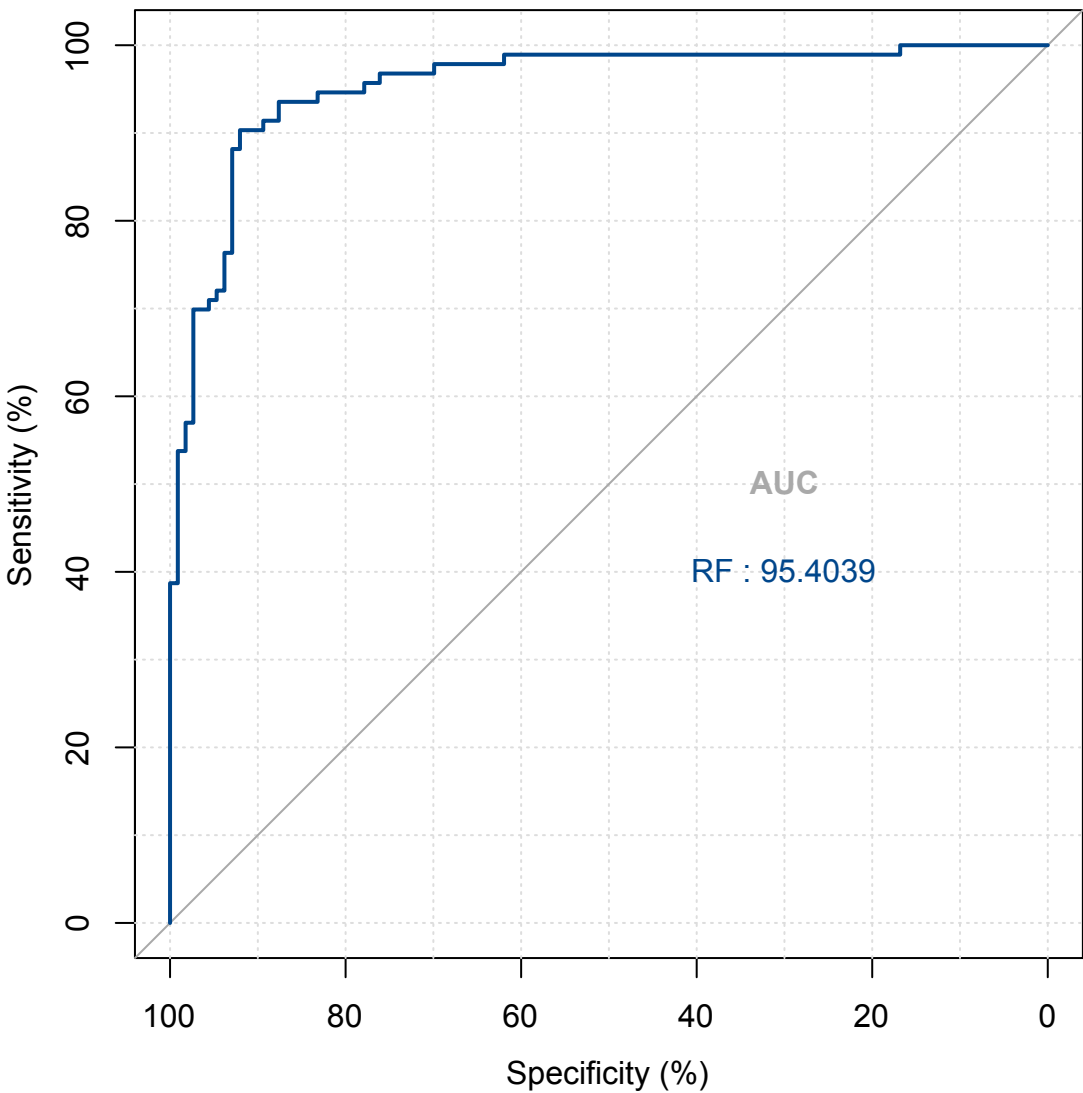

Supplement: Supplementary file 17 [file DataSheet_3.zip › Predictive model/meta-cohort-Phenotype C.pdf]

# ROC Plot

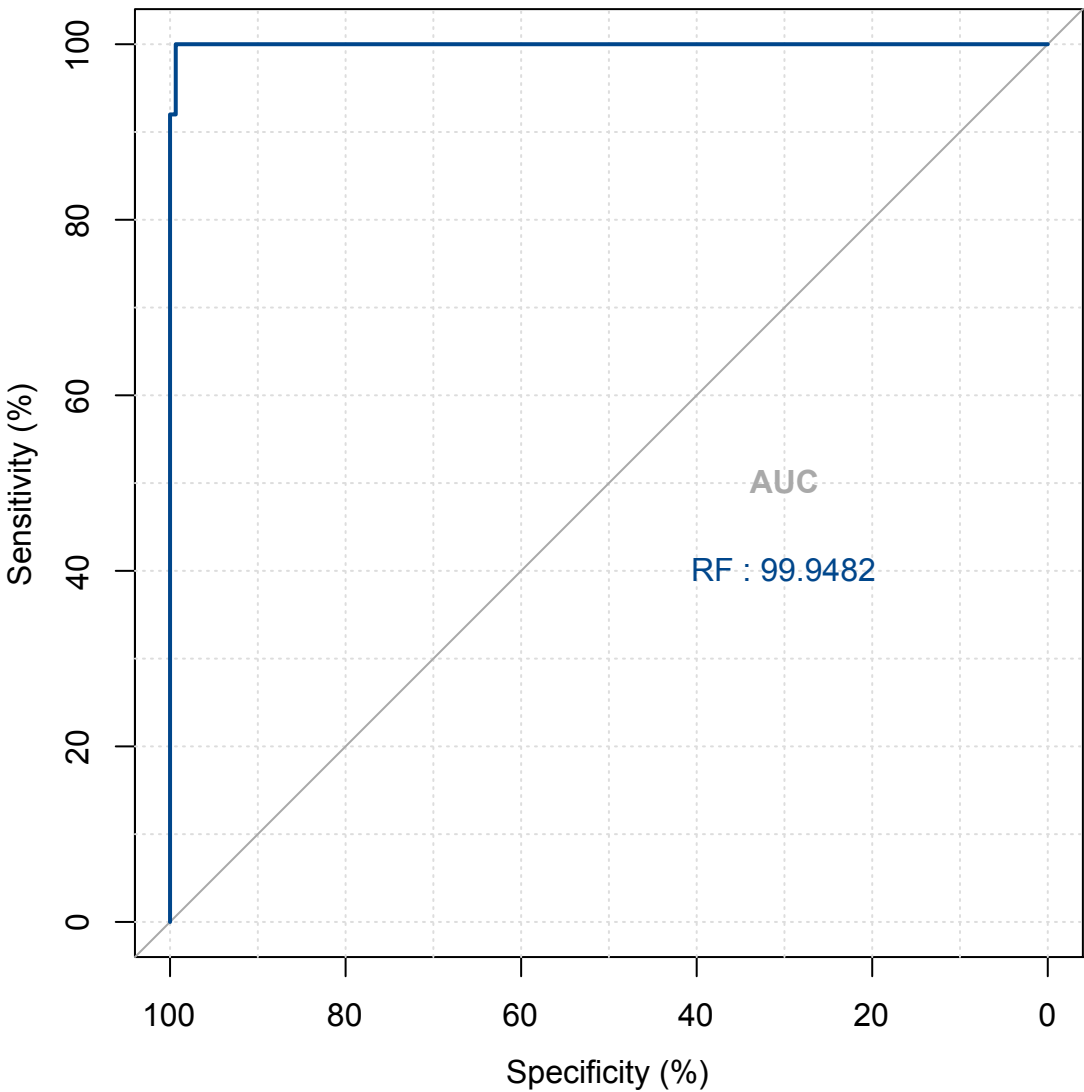

Supplement: Supplementary file 17 [file DataSheet_3.zip › Predictive model/Training-cohort-Phenotype A.pdf]

# ROC Plot

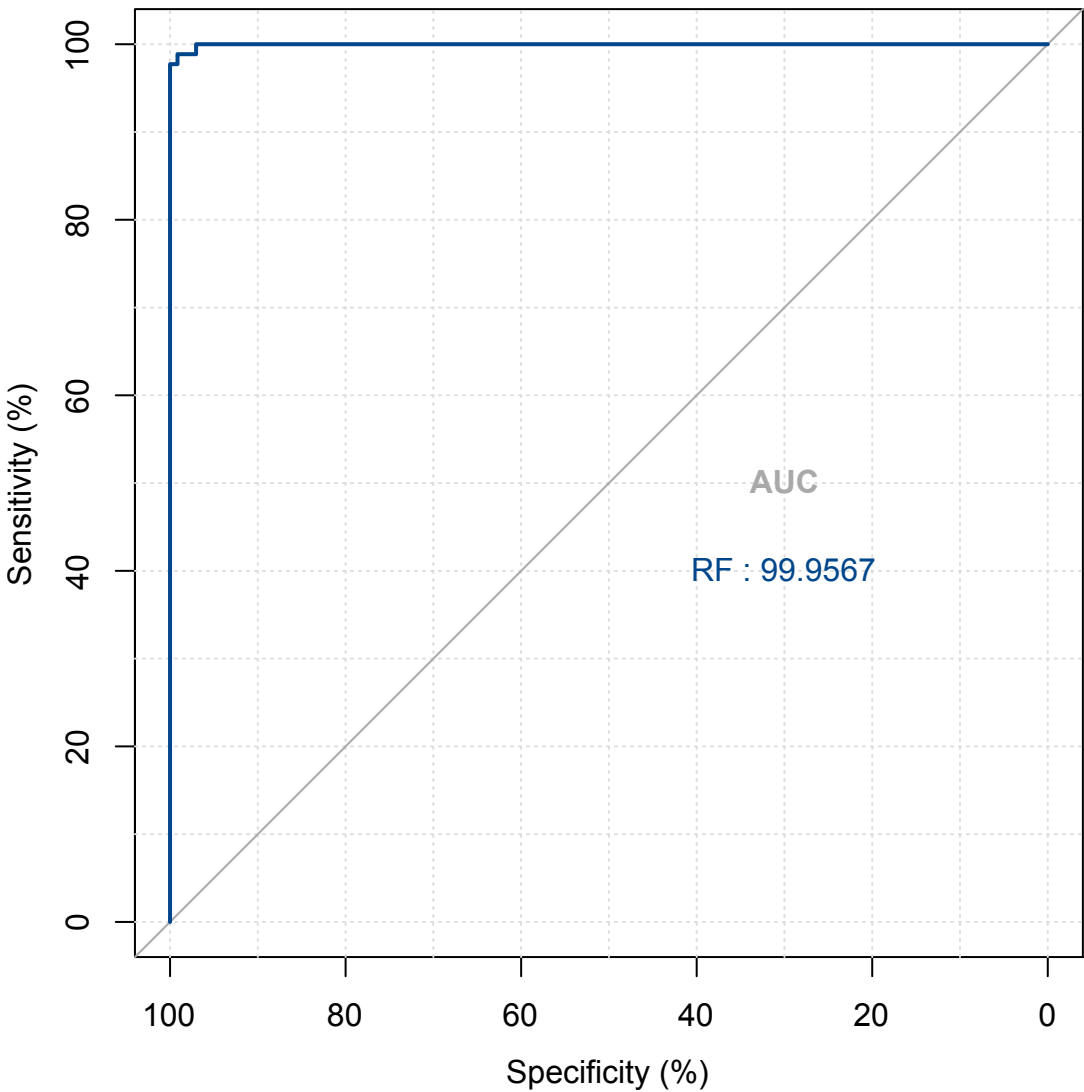

Supplement: Supplementary file 17 [file DataSheet_3.zip › Predictive model/Training-cohort-Phenotype B.pdf]

# ROC Plot

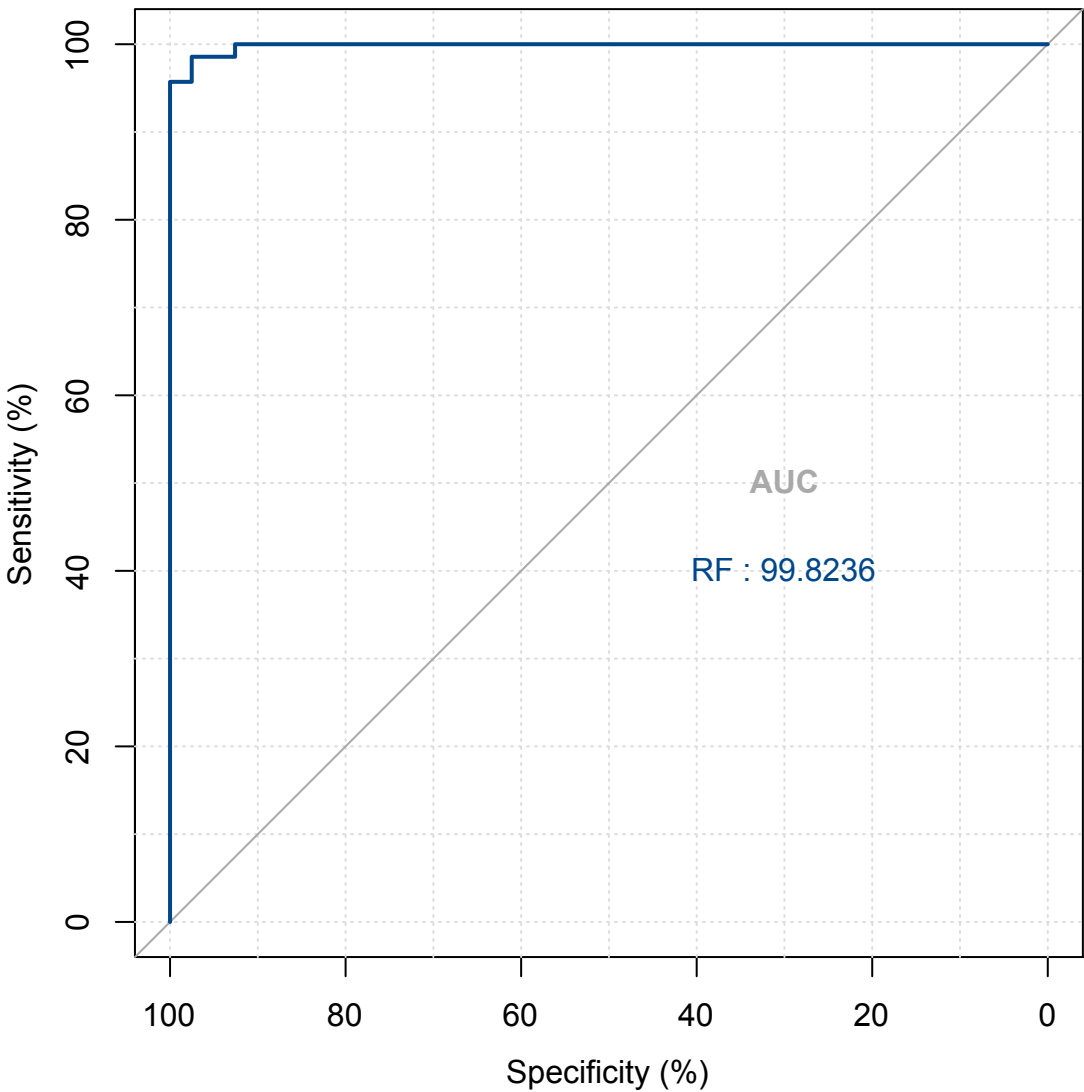

Supplement: Supplementary file 17 [file DataSheet_3.zip › Predictive model/Training-cohort-Phenotype C.pdf]

# ROC Plot

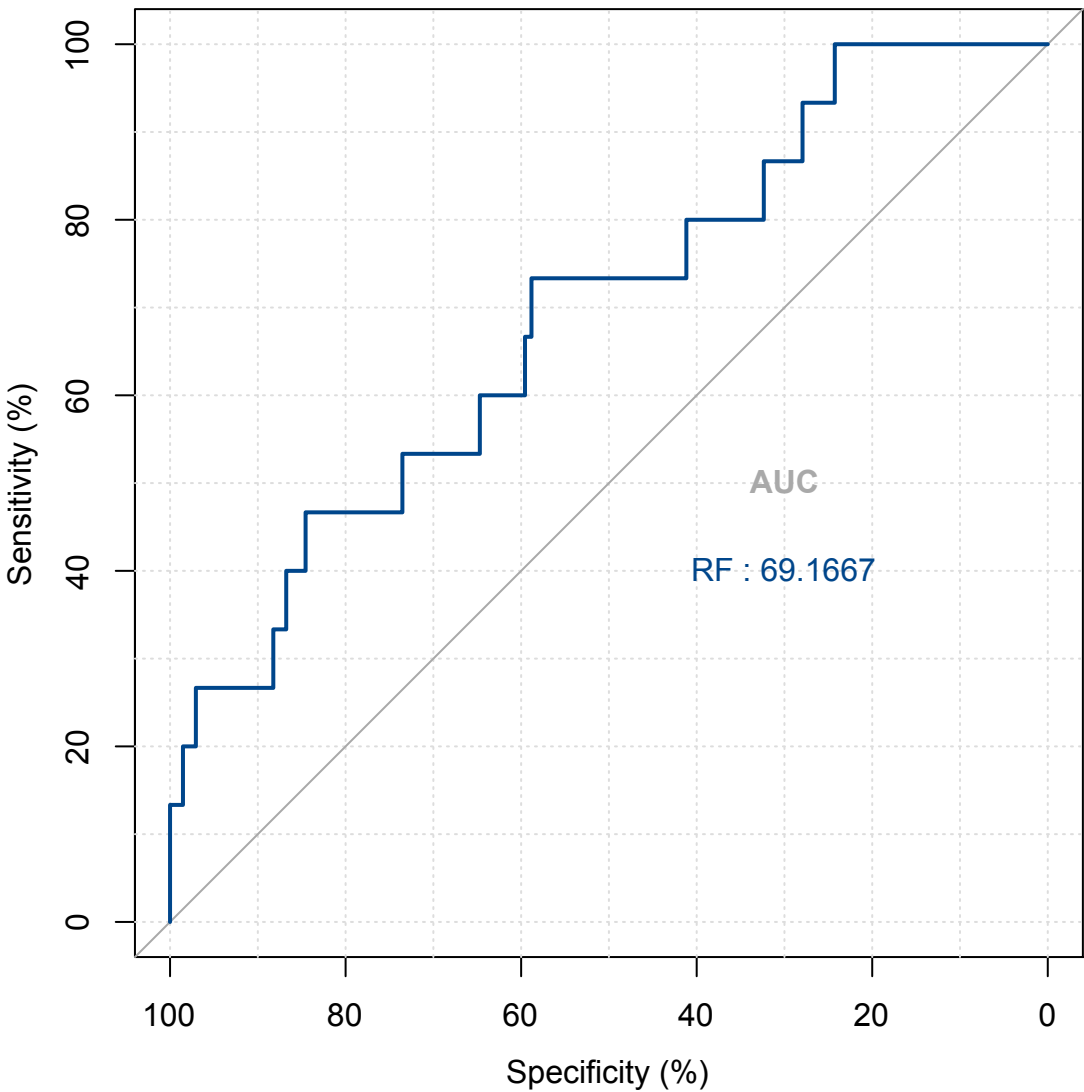

Supplement: Supplementary file 17 [file DataSheet_3.zip › Predictive model/Validation-cohort-Phenotype A.pdf]

# ROC Plot

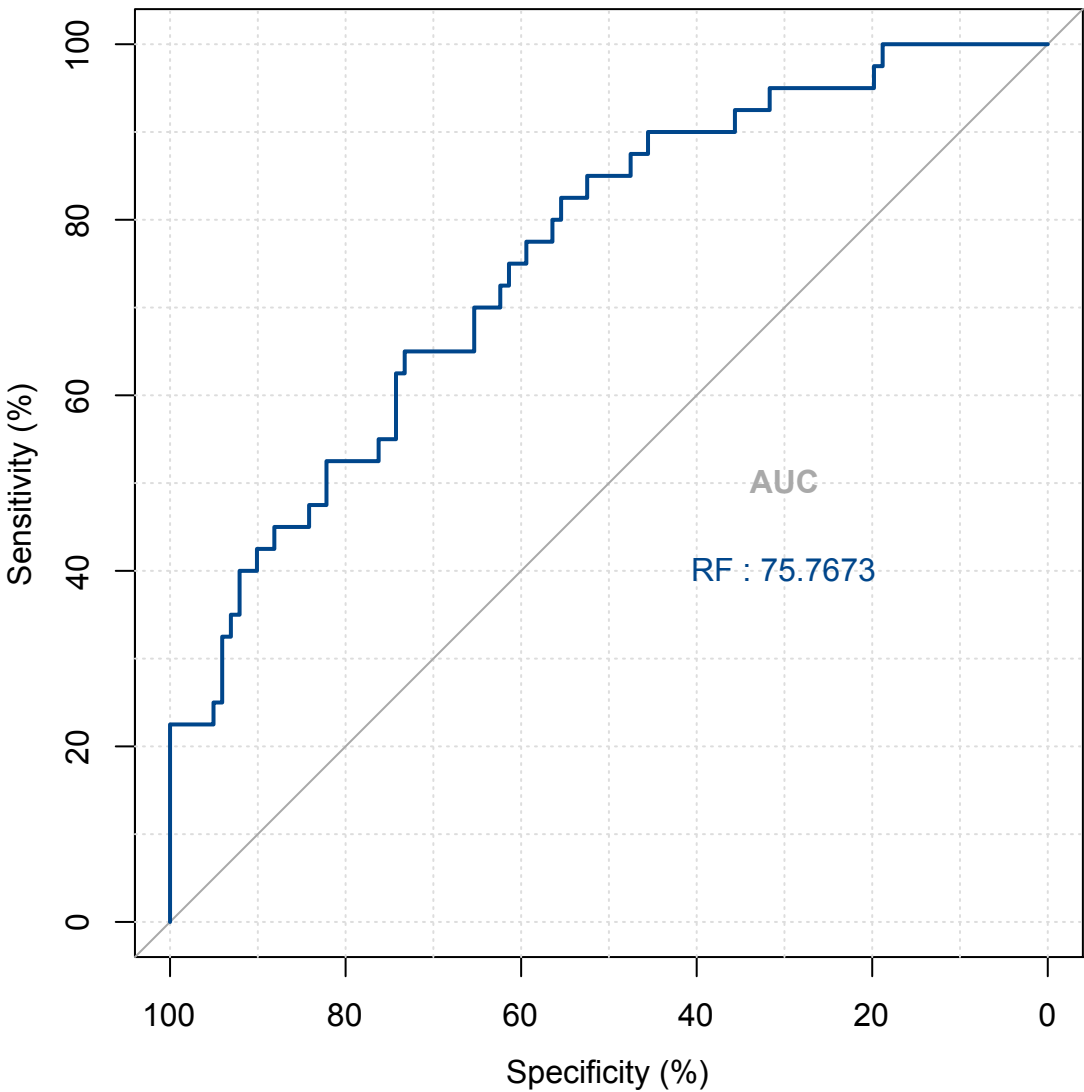

Supplement: Supplementary file 17 [file DataSheet_3.zip › Predictive model/Validation-cohort-Phenotype B.pdf]

# ROC Plot

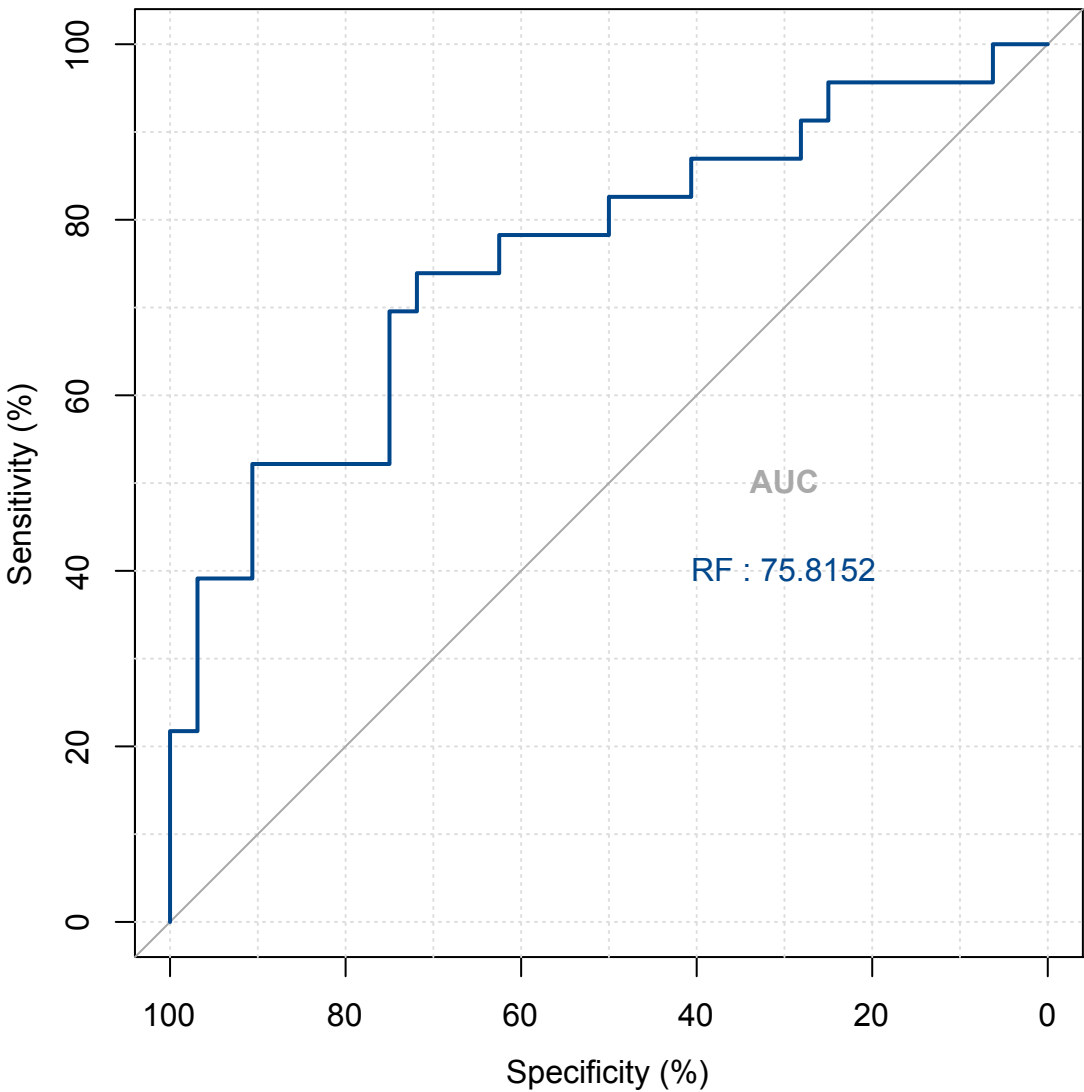

Supplement: Supplementary file 17 [file DataSheet_3.zip › Predictive model/Validation-cohort-Phenotype C.pdf]

Time-Dependent ROC

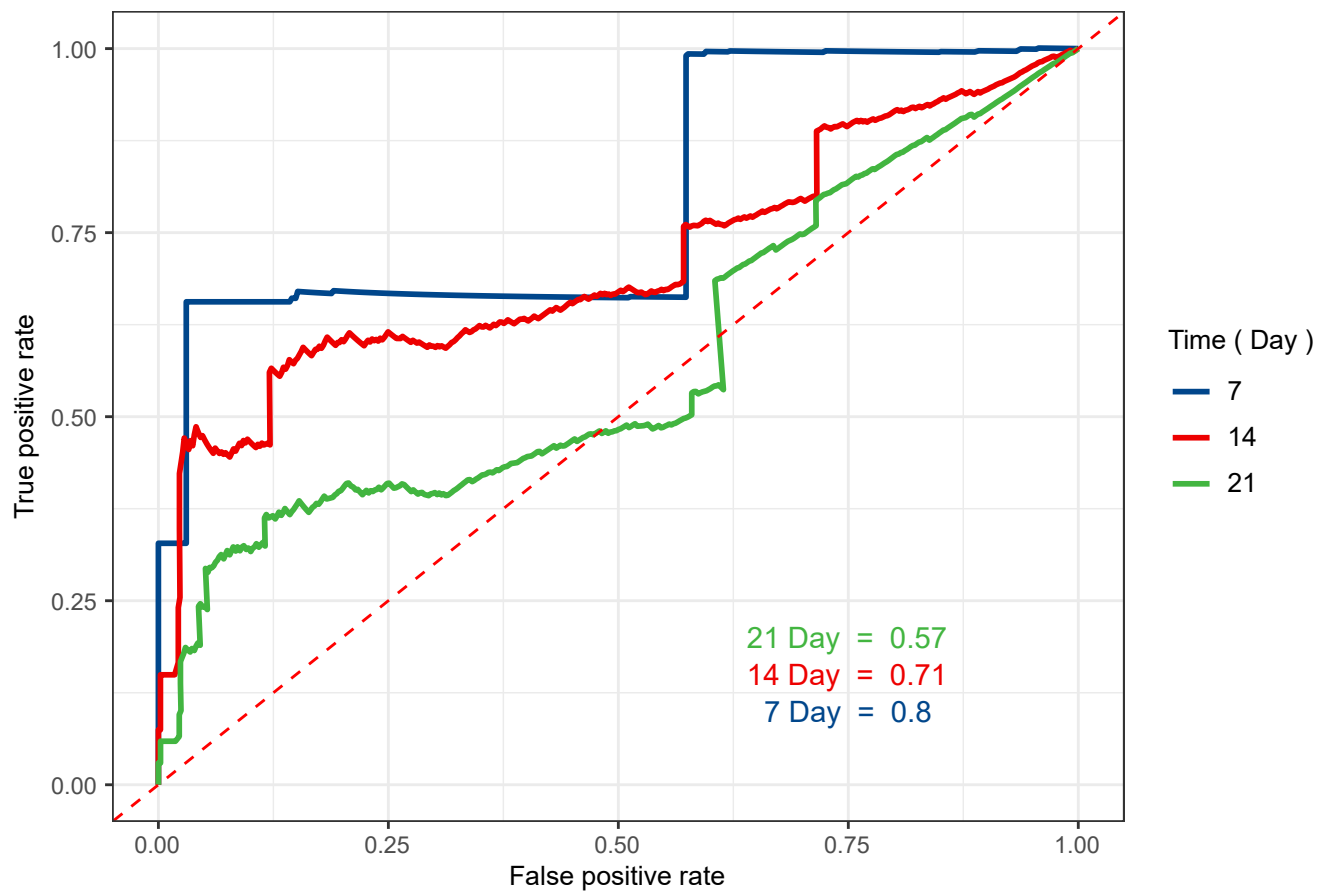

Supplement: Supplementary file 17 [file DataSheet_3.zip › Prognostic model/meta-cohort-Phenotype B.pdf]

Time-Dependent ROC

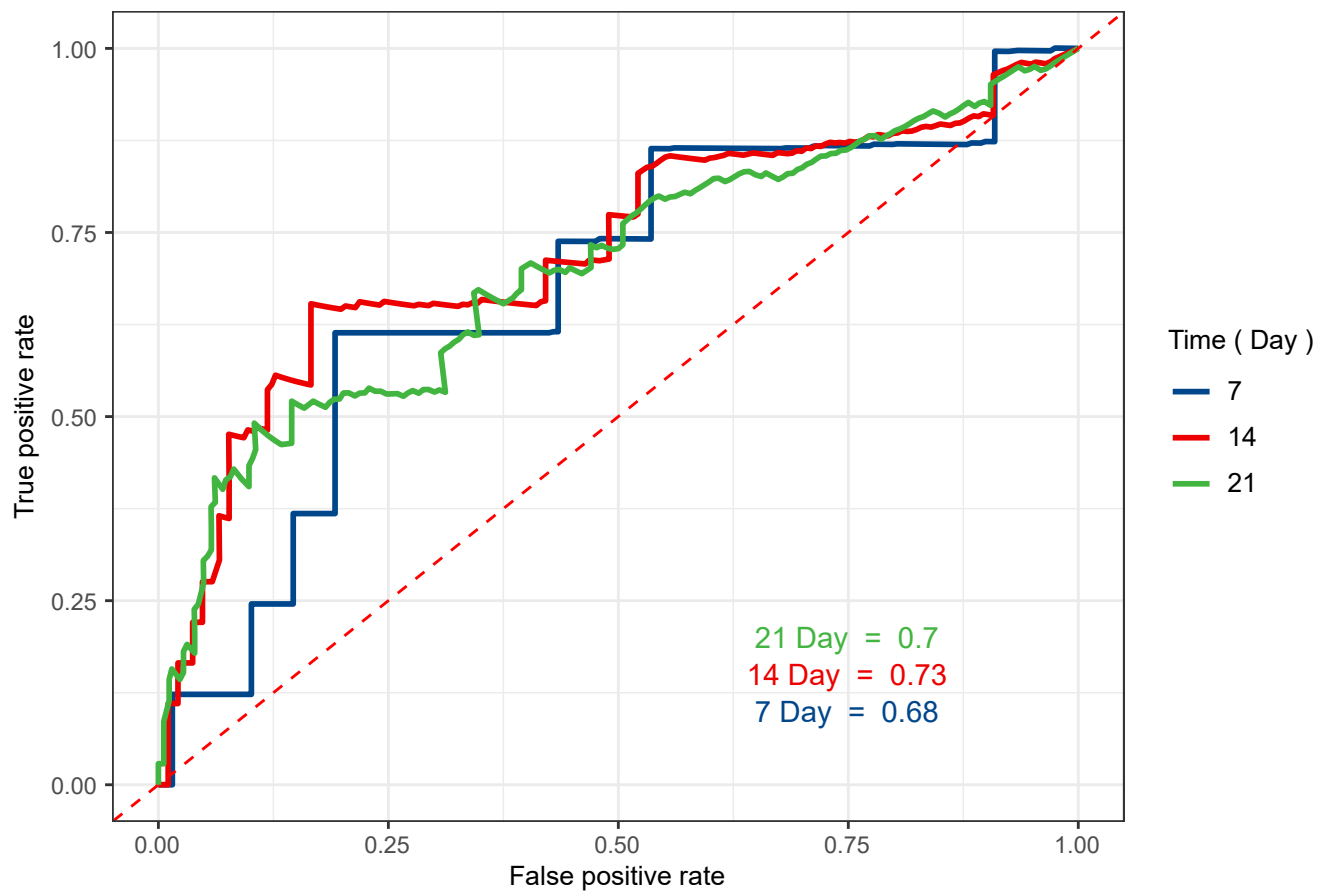

Supplement: Supplementary file 17 [file DataSheet_3.zip › Prognostic model/meta-cohort-Phenotype C.pdf]

Time-Dependent ROC

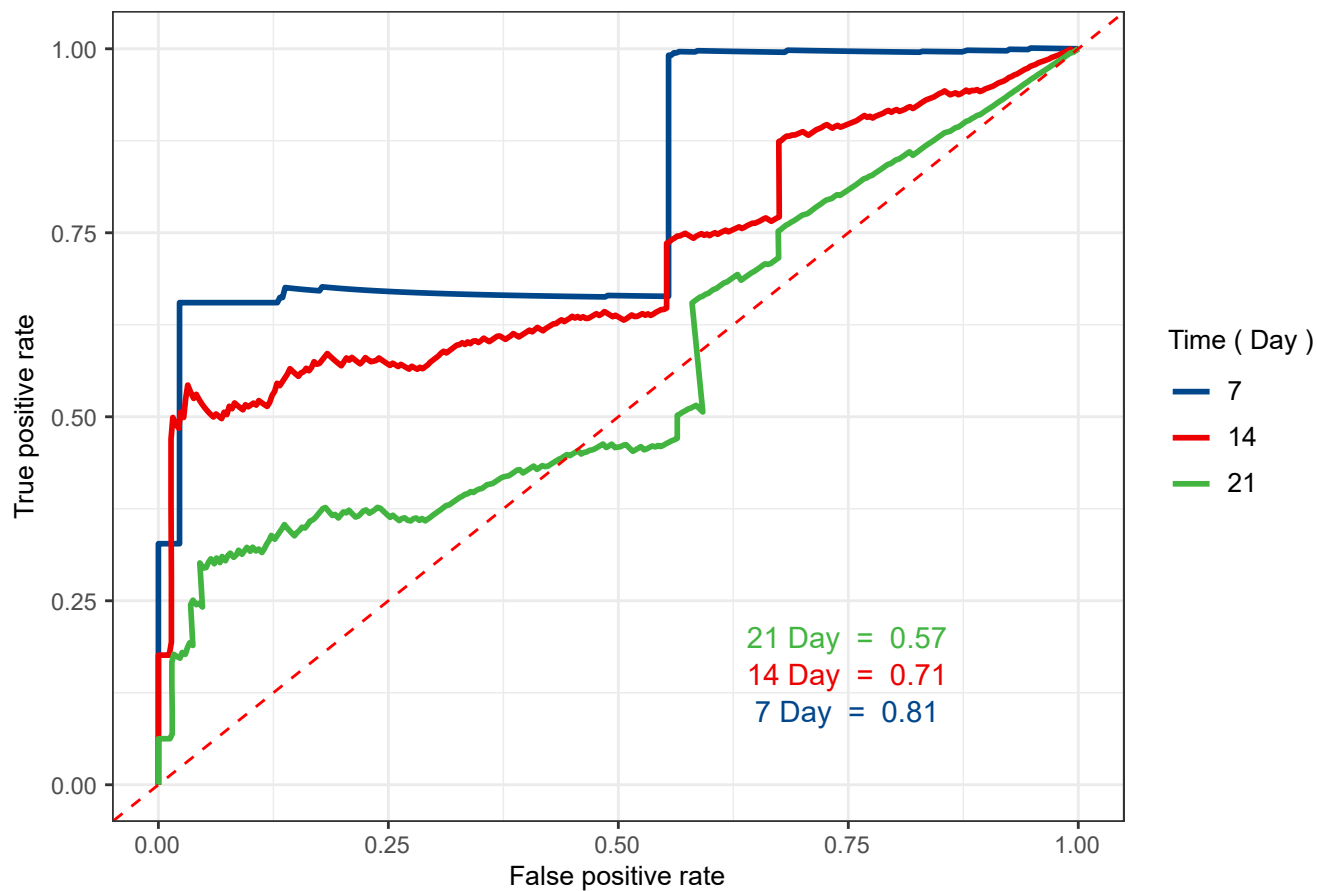

Supplement: Supplementary file 17 [file DataSheet_3.zip › Prognostic model/Training-cohort-Phenotype B.pdf]

Time-Dependent ROC

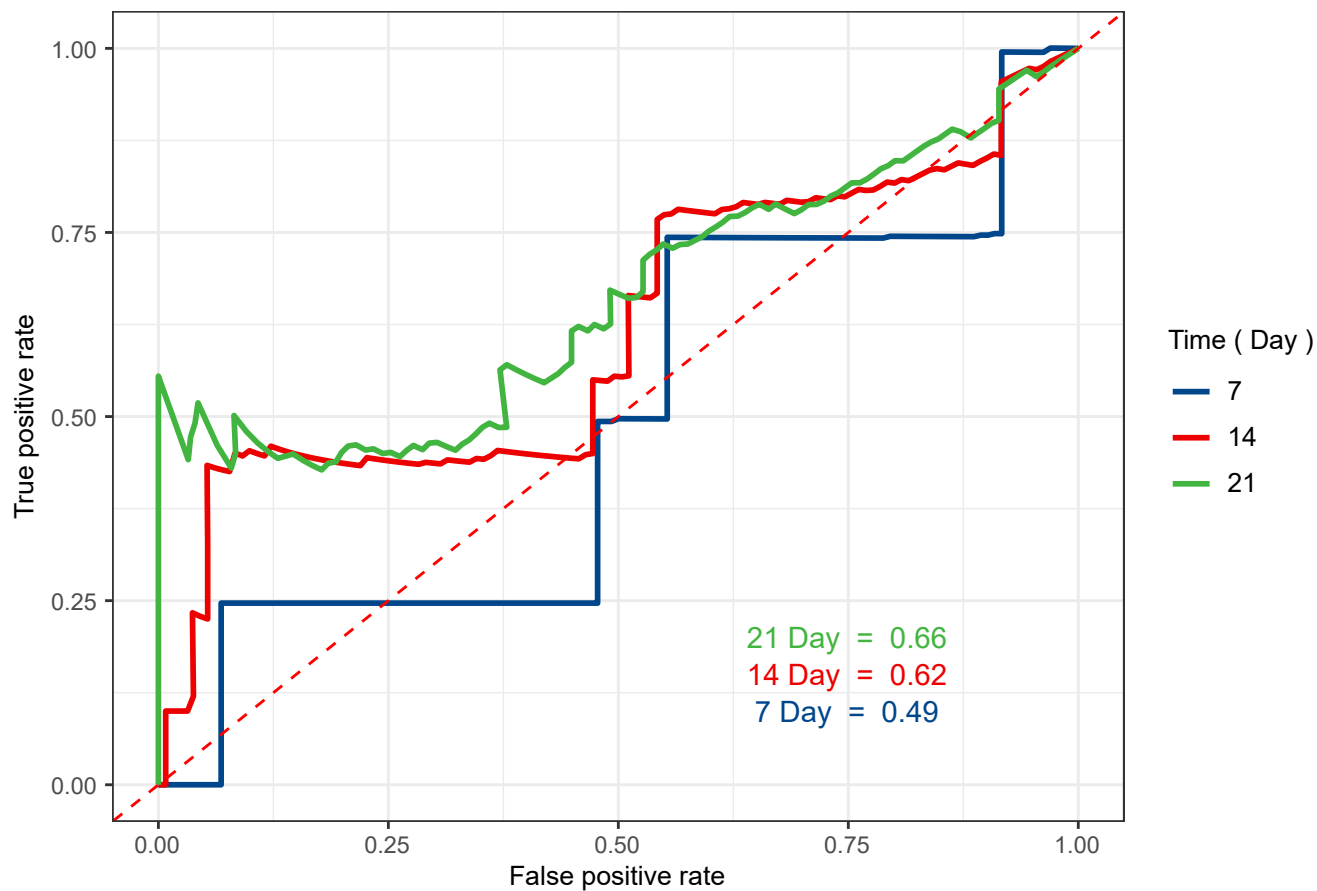

Supplement: Supplementary file 17 [file DataSheet_3.zip › Prognostic model/Training-cohort-Phenotype C.pdf]

Time-Dependent ROC

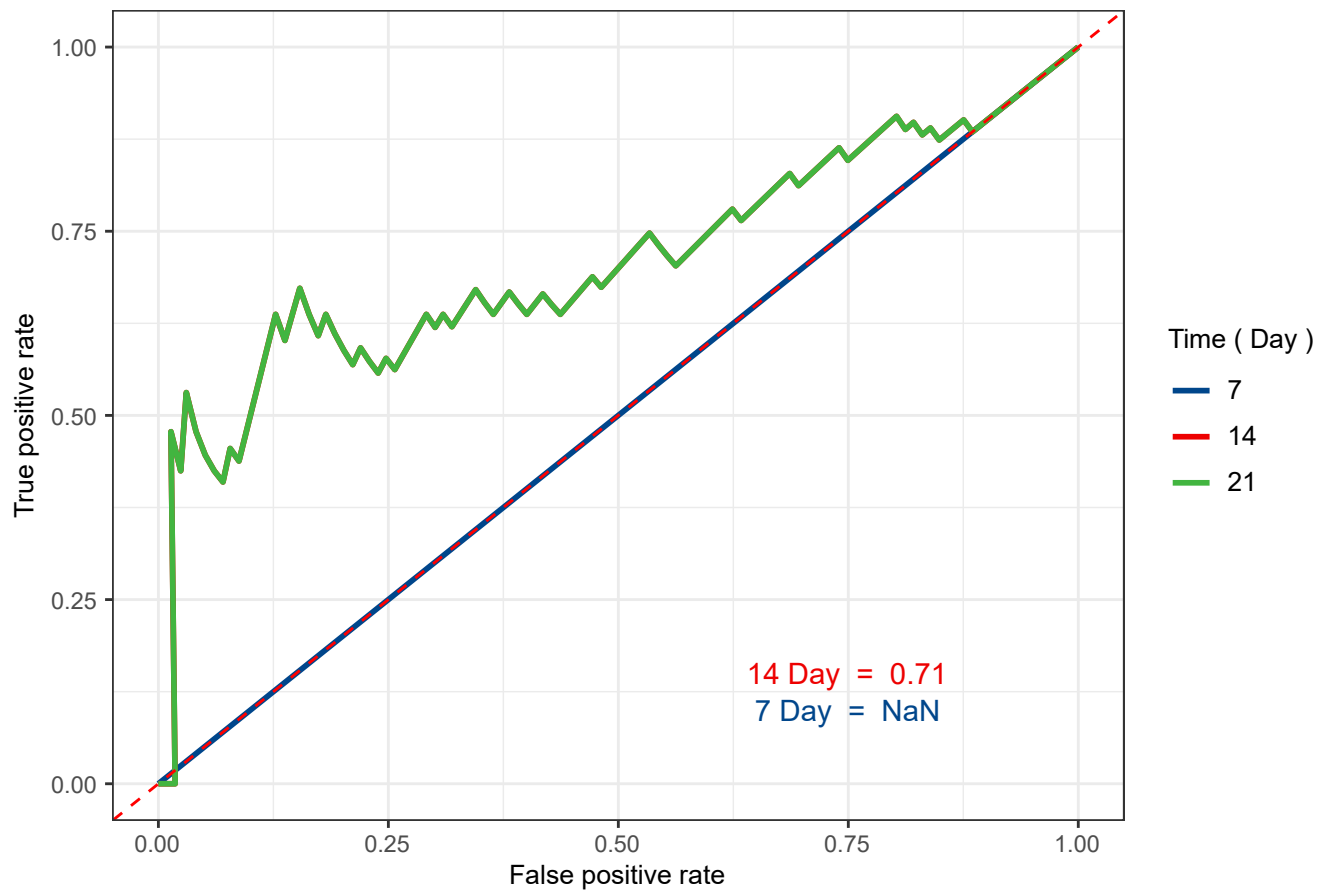

Supplement: Supplementary file 17 [file DataSheet_3.zip › Prognostic model/Validation-cohort-Phenotype B.pdf]

Time-Dependent ROC

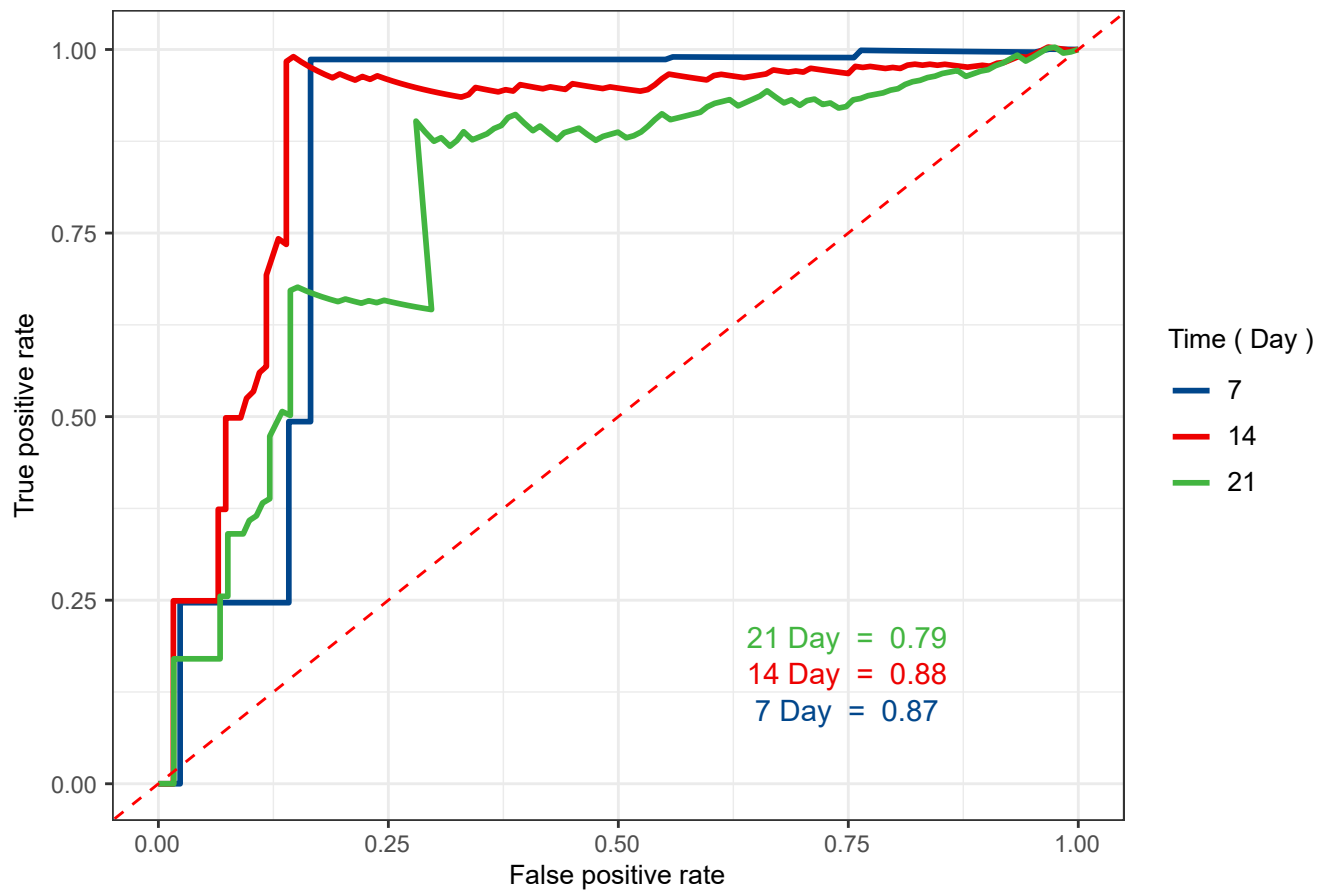

Supplement: Supplementary file 17 [file DataSheet_3.zip › Prognostic model/Validation-cohort-Phenotype C.pdf]
